# Magnetic COFs as satisfactory support for lipase immobilization and recovery to effectively achieve the production of biodiesel by maintenance of enzyme activity

**DOI:** 10.1186/s13068-021-02001-0

**Published:** 2021-07-14

**Authors:** Zi-Wen Zhou, Chun-Xian Cai, Xiu Xing, Jun Li, Zu-E. Hu, Zong-Bo Xie, Na Wang, Xiao-Qi Yu

**Affiliations:** 1 Key Laboratory of Green Chemistry and Technology (Ministry of Education), College of Chemistry, Sichuan University, Chengdu, 610064 People’s Republic of China; 2 Jiangxi Province Key Laboratory of Synthetic Chemistry, School of Chemistry, Biology and Material Science, East China University of Technology, Nanchang, 330013 People’s Republic of China

## Abstract

**Background:**

Production of biodiesel from renewable sources such as inedible vegetable oils by enzymatic catalysis has been a hotspot but remains a challenge on the efficient use of an enzyme. COFs (Covalent Organic Frameworks) with large surface area and porosity can be applied as ideal support to avoid aggregation of lipase and methanol. However, the naturally low density limits its application. In this work, we reported a facile synthesis of core–shell magnetic COF composite (Fe_3_O_4_@COF-OMe) to immobilize RML (*Rhizomucor miehei* lipase), to achieve its utilization in biodiesel production.

**Result:**

This strategy gives extrinsic magnetic property, and the magnetic COFs is much heavier and could disperse in water medium well, facilitating the attachment with the enzyme. The resultant biocomposite exhibited an excellent capacity of RML due to its high surface area and fast response to the external magnetic field, as well as good chemical stability. The core–shell magnetic COF-OMe structure not only achieved highly efficient immobilization and recovery processes but also maintained the activity of lipase to a great extent. RML@Fe_3_O_4_@COF-OMe performed well in practical applications, while free lipase did not. The biocomposite successfully achieved the production of biodiesel from *Jatropha curcas* Oil with a yield of about 70% in the optimized conditions.

**Conclusion:**

Magnetic COFs (Fe_3_O_4_@COF-OMe) for RML immobilization greatly improved catalytic performance in template reaction and biodiesel preparation. The magneticity makes it easily recovered and separated from the system. This first successful attempt of COFs-based immobilized enzyme broadened the prospect of biodiesel production by COFs with some inspiration.

**Supplementary Information:**

The online version contains supplementary material available at 10.1186/s13068-021-02001-0.

## Background

With the character of biodegradability, biodiesel from renewable sources such as vegetable oils and animal fat produced by enzymatic transesterification has attracted the interest of scientists [1, 2]. Immobilized lipases received incredible interest among the biotechnology community for the production of biodiesel with the advantages of absence of side reactions, low energy requirement, and free pollutions, compared to basic chemical catalysts [3]. A range of carriers such as microemulsion-based organogels (MBGs), [4] carbon nanotube [3, 5], silica nanoparticles [6], and organic polymers [7] have been successfully utilized. However, such common supports usually affect the enzymatic activity and the relatively small surface area limits its loading capacity.

In recent years, porous materials have been developed and applied to integrate enzymes based on their excellent features of high surface area and porosity which facilitate the immobilization process and preservation of biomolecules [8]. Metal–Organic Frameworks (MOFs) with high surface area, pore channels, and chemical functionality [9–11] has been reported to entrap lipase for biodiesel [12–14]. However, MOFs is chemically unstable and toxic due to the metal ions, since some are water-sensitive or easily digested [15–17]. In addition, the leakage of unwanted metal ions may harm biomolecule conformation. As a sister material of MOFs, Covalent-Organic Frameworks (COFs), without toxic metal ion, is highly desirable because of long-term water/chemistry stability [18, 19]. This emerging porous nanomaterial are prepared by linkage of organic building units covalently into extended structures to make crystalline materials, whose backbones are composed of light elements (B, C, N, O, Si) [20, 21]. Since the constituents of COFs do not undergo significant change when covalently linked, the structures of resulting COFs with desired composition, pore size, and aperture could be predicted and prepared by the programming of monomers [20, 22]. Its tunability and stability enable its application in gas storage [23, 24], sensing [25, 26], and catalysis [27, 28], as well as immobilization of enzyme. This is because COFs could provide appropriate abundant micro and mesopore channels, affording large surface area for infiltrating biomolecules. Through design, the good matched pore size could impede the aggregation of enzymes and facilitate the rapid transportation of reagents at the same time [29]. There are also specific sites on the interface for weak interaction or covalent binding [30, 31], so that the immobilization process varies with a practical need [17].

Since Kandambeth et al. reported the first example to utilize COFs for enzyme immobilization [32], a few COF-based immobilized enzymes are reported for applications, such as the kinetic resolution of secondary alcohols by Sun et al. (TPB-DMTP-COF@Lipase PS) [33] and chiral separation by Zhang et al. (lysozyme covalently immobilized on COF 1) [34]. As one of the common substrates methanol has an adverse effect on the lipase [35], with large surface area and porosity, COFs can be an ideal carrier for lipase adsorption and reagents transportation, which impedes the aggregation of enzyme and the effect of methanol, indicating a potential utility for enzyme immobilization and biodiesel production. However, there are also some challenges for the highly efficient use of COFs as a carrier. One of the matters is attributed to the backbone consisted of light elements. For one thing, the naturally low density [36] hinders its dispersion in water solution, which is not conducive to the enzyme enrichment on the support. For another, the strong electrostatic interaction between nanoparticles and containers (such as plastic tubes) makes it adhere to the surface, which is not convenient to gather and preserve. Therefore, it has not been reported for COFs-based immobilized lipase to achieve the transition of biodiesel, and these inconveniences hinder its applied research in industrial production.

**Scheme 1:**
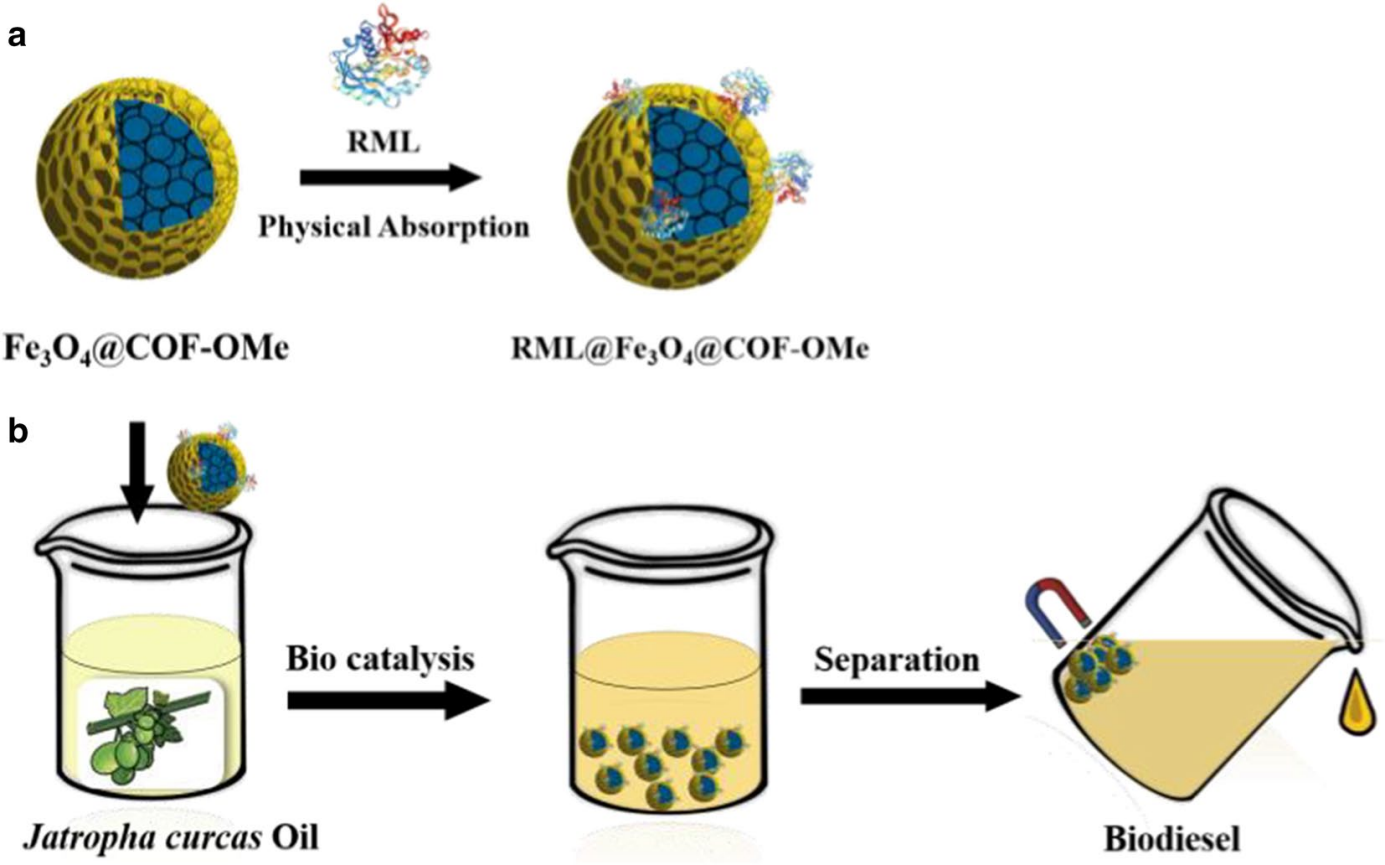
Schematic representation of **a** core–shell magnetic nanoparticles and immobilization process and **b** its application in production of biodiesel

Our group has reported several works about the immobilized enzyme in applications such as biomass conversion and biodiesel production [37–39]. During these processes, we have introduced Fe_3_O_4_ nanoparticles to facilitate recovery. Thus, we conceived a magnetic strategy to give extrinsic magnetic property to COFs to overcome these factors mentioned before. The resultant composite is much heavier and could disperse in water medium well, facilitating the attachment with the enzyme. The magnetic COFs can be recycled by an external magnetic fields and reduce the aggregation of lipase and influence of methanol [40–43], which lays a good foundation for its efficient industrial production of biodiesel. Here, we reported core–shell magnetic COFs (Fe_3_O_4_@COF-OMe), in which Fe_3_O_4_ nanoparticles are wrapped by COF-shell. This composite can be dispersed in PBS buffer and recovered easily due to the introduction of magneticity. The prepared composite is used to immobilized RML (*Rhizomucor miehei* lipase, EC 3.1.1.3, a kind of lipase, triacylglycerol hydrolases) by physical absorption, with a high enzyme uptake capacity and good maintenance of activity in harsh conditions. The resultant immobilized RML finally achieved the production of biodiesel from a renewable source, *Jatropha curcas* Oil (inedible vegetable oil) with a satisfactory yield after optimizing the conditions of template reactions (Scheme 1).

## Results and discussion

### Preparation and characterization of Fe_3_***O***_***4***_@COF-OMe nanoparticles and immobilized RML

**Fig. 1:**
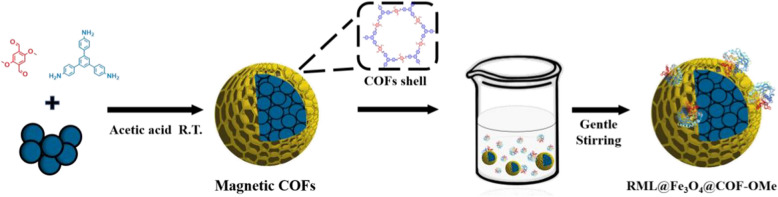
Synthesis of core–shell magnetic nanoparticles and immobilization process

The facile synthesis of magnetic core–shell COFs is based on the room-temperature synthesis of COF-OMe (Additional file 1: Figs. S1–S4). The detailed preparation and immobilization process is illustrated in Fig. 1, which involved two main steps: (1) coprecipitation synthesis of magnetic Fe_3_O_4_ nanoparticles and rapid room-temperature synthesis of the core–shell structured magnetic Fe_3_O_4_@COF-OMe composites in a one-pot process by mixing Fe_3_O_4_ nanoparticles (30 mg, 0.13 mmol) as the magnetic core and 2,5-dimethoxyterephthalaldehyde (DMTP, 0.24 mmol) and 1,3,5-tris(4-aminophenyl)-benzene (TPB, 0.16 mmol) as building units of COF-OMe in the acetonitrile according to the result of morphology (Additional file 1: Fig. S5). (2) immobilization process of RML by physical absorption in PBS buffer. The as-prepared biocomposites could be applied in the production of biodiesel.

**Fig. 2:**
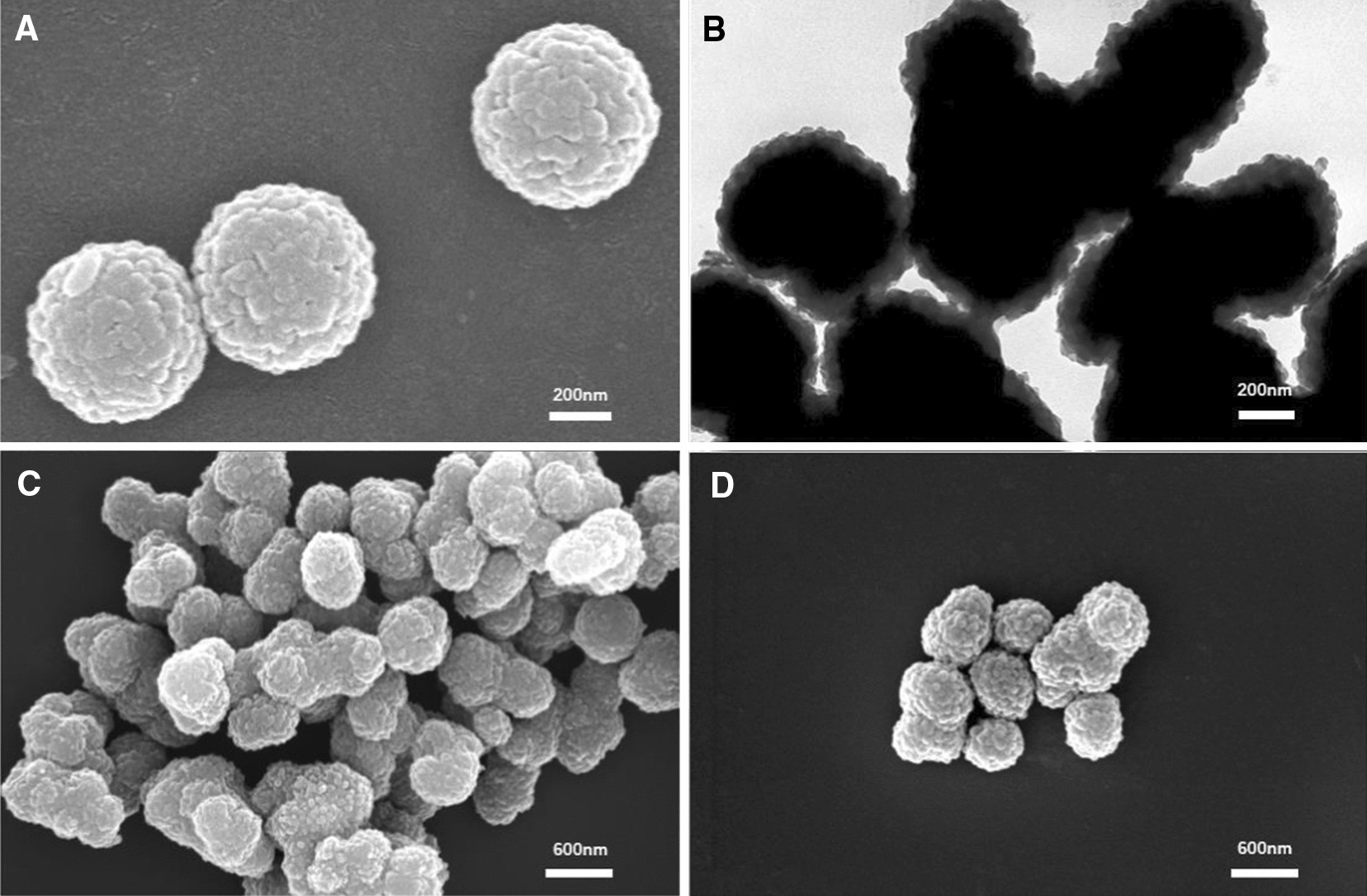
SEM image of **A** COF-OMe; TEM image (**B**) and SEM image (**C**) of core–shell structure Fe_3_O_4_@COF-OMe; SEM image (D) of RML@Fe_3_O_4_@COF-OMe

The morphologies of COF-OMe, Fe_3_O_4_@COF-OMe, and RML@Fe_3_O_4_@COF-OMe are verified by scanning electron microscopy (SEM) and transmission electron microscopy (TEM), as shown in Fig. 2. It can be seen that COF-OMe has good dispersity and exhibits a uniform nanosphere structure (Fig. 2A) with a size of 500–600 nm. The core–shell structure of Fe_3_O_4_@COF-OMe with the thickness of the COF shell about 70 nm is confirmed by the TEM image (Fig. 2B). The Fe_3_O_4_@COF-OMe particles display similar spherical morphology as COF-OMe (Fig. 2C). In addition, after enzyme immobilization, the composite morphology remains unchanged (Fig. 2D).

Fourier transforms infrared (FT-IR) spectroscopy is carried out to prove that the successful synthesis of Fe_3_O_4_@COF-OMe. As shown in Additional file 1: Figure S6, the FTIR spectrum of Fe_3_O_4_ contains a band at 579 cm^−1^, which is assigned to characteristic Fe–O–Fe stretch. The characteristic absorption bands at 1610 cm^−1^ assigned to the C=N stretch mode observed in the curve of Fe_3_O_4_@COF-OMe means the successful synthesis of COF-OMe by condensation of aldehydes and amines. Along with the characteristic Fe–O–Fe stretch found in the curve of biocomposite, the combination of COF-shell and magnetic Fe_3_O_4_ was proved, demonstrating the successful preparation of Fe_3_O_4_@COF-OMe. [40].

**Fig. 3:**
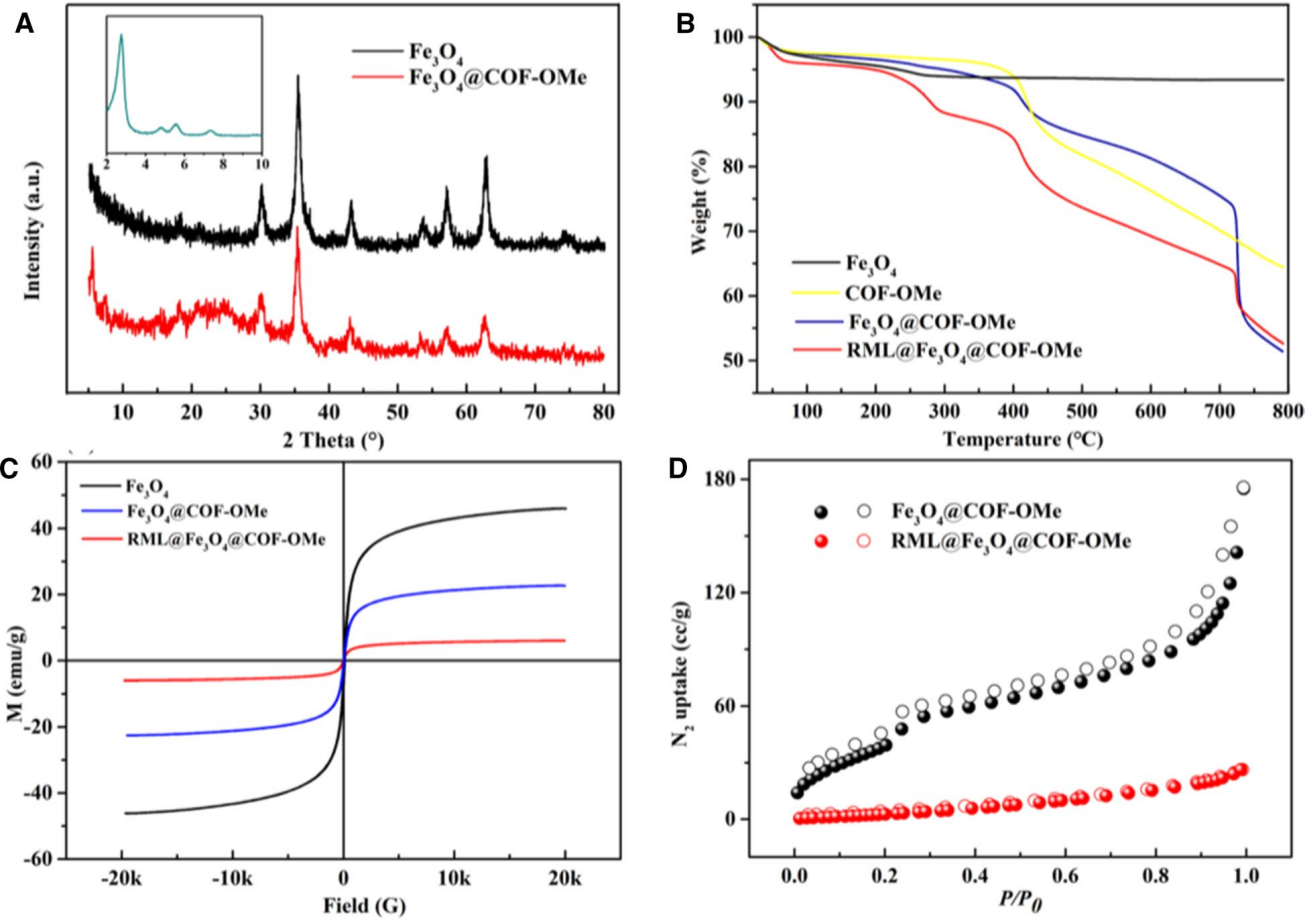
**A** PXRD patterns of Fe_3_O_4_ and Fe_3_O_4_@COF-OMe particles (the inset shows the patterns of crystalline COF-OMe). **B** TGA curves, **C** magnetic hysteresis curve and **D** N_2_ adsorption–desorption isotherms of the magnetic support Fe_3_O_4_@COF-OMe and the immobilized enzyme RML@Fe_3_O_4_@COF-OMe

The crystalline structure of Fe_3_O_4_@COF-OMe is examined by PXRD patterns (Fig. 3A). XRD image exhibits 6 peaks with 2*θ* at 30.12°, 35.42°, 43.18°, 53.64°, 56.96°, and 62.60°, corresponding to (220), (311), (400), (422), (511) and (440) [40], which matches well with magnetite, indicating that the Fe_3_O_4_@COF-OMe are well crystallized after coating COFs. As to COFs, the characteristic peak appears about 2.0° of 2*θ* (Cu Kα1), attributed to the (100) facet of a primitive hexagonal lattice [29]. This is found in the PXRD image of COF-OMe pattern, shown inset. The peak at 2.75° is attributed to the plane (100) of COF-OMe. The other planes, like (110), (200), (210), (220) corresponds to peaks at 4.8°, 5.5°, 7.3° and 9.2° [44]. This pattern confirms the formation of the crystalline form of COF-OMe. These successful and facile preparation represents it an alternative way of traditional synthesis of them, which provides guidance for the exploration of other COFs.

Thermogravimetry analysis (TGA) expounds on the thermal stability and different components of biocomposites, as shown in Fig. 3B and Additional file 1: Fig. S7 (DTG). For COF-OMe, there is a distinct decrease in weight that occurs at 300–400 ℃, which means its structure begins to disintegrate. In other words, a long plateau under 419 °C demonstrates the high thermal stability of COF-OMe. As for RML@Fe_3_O_4_@COF-OMe, the weight loss at about 280 °C can be attributed to the removal of lipase (Additional file 1: Figure S8). The two parts of mass losses occurred at 48 °C and 410 °C is consistent with it of bare Fe_3_O_4_ (4% at 48 °C) and COF-OMe (12% at 410 °C), respectively. The sharp weight loss at over 700 °C may due to the reaction between melt COF-OMe and Fe_3_O_4_ of core–shell structure. In a word, the support Fe_3_O_4_@COF-OMe displays such satisfactory thermal stability as COF-OMe, where the TG curve runs smoothly under 400 °C. At the same time, the core–shell structure doesn’t react mutually under 700 °C, which means it is qualified to be a good carrier of an enzyme.

The magnetic property of these nanospheres is characterized by a vibrating sample magnetometer (VSM). The magnetic hysteresis curve (Fig. 3C) of nanomagnetic Fe_3_O_4_ has an excellent magnetic property, with a saturated magnetization value of 46.07 emu g^−1^. There is a drop observed in Fe_3_O_4_@COF-OMe (~ 20 emu g^−1^) and RML@Fe_3_O_4_@COF-OMe (~ 6 emu g^−1^), which are attributed to the loading of COF shell and enzyme. Despite this, rapid aggregation of biocomposites from the suspension is obtained with the help of an external magnet, which could reduce the desorption of the enzyme by centrifugation in this way.

Nitrogen sorption isotherms measured at 77 K indicates the BET surface of Fe_3_O_4_@COF-OMe decreases from 232 cm^2^ g^−1^ to 28 cm^2^ g^−1^ after immobilization of RML (Fig. 3D). The pore-size distribution analyses of Fe_3_O_4_@COF-OMe and lipase@Fe_3_O_4_@COF-OMe calculated by the density functional theory have shown that both of the samples have a pore size centered at about 3.1 nm, whereas the pore volume drops from 0.223 cc g^−1^ to 0.036 cc g^−1^ after RML absorption (Additional file 1: Figure S9 and Table S1). The result indicates the successfully loading of lipase, and it suggests that the magnetic COFs may serve as a promising carrier for lipase immobilization.

To further verify the distribution of RML on the support, the fluorescein-labelled enzyme is an optical way to prove its existence and determine its distribution. Fluorescent probe fluorescein isothiocyanate (FITC) is used to label the enzyme molecules (green) generally [43, 45]. However, it is not available to use in RML@COF-OMe in this work. This is because the support, COF-OMe itself, is fluorescent. Under an excitation *λ* = 488 nm (the parameter of FITC-labelled protein), the long emission at *λ* = 490–690 nm is got by the COF-OMe itself, which interferes with the detection of the FITC-labelled enzyme, so it is incapable to prove the existence of enzyme on the surface of the carrier and determine its distribution (Additional file 1: Figure S10). In this case, Rhodamine B isothiocyanate (RBITC)-labeled RML was prepared. The RBITC-labelled RML (red) is present throughout Fe_3_O_4_@COF-OMe (green), which is observed by CLSM analysis at excitation wavelengths of 488 nm for Fe_3_O_4_@COF-OMe and 543 nm for RBITC-RML, demonstrating that the enzyme accommodated in this composite (Additional file 1: Figure S11).

### The optimization of the immobilization process

**Fig. 4:**
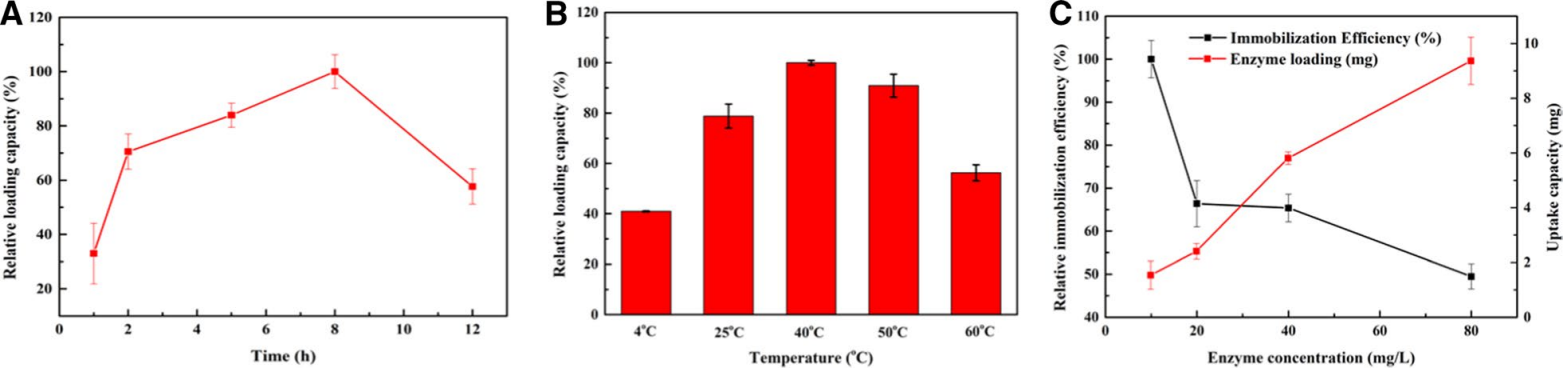
Effect of **A** time; **B** temperature; and **C** RML concentration on the performance of RML@ Fe_3_O_4_@COF-OMe in immobilization process

RML is immobilized on the carrier by physical absorption and different conditions of immobilization will affect the loading of enzymes. Then, the effect of time, concentration of lipase, and temperature were studied during the immobilization process. The enzyme loading of Fe_3_O_4_@COF-OMe increased with the time at the beginning (Fig. 4A), and decreased after 8 h. It can be explained that long-term shaking caused leakage of lipase after absorption saturation. The temperature made an influence on both immobilization efficiency and activity of RML. In the immobilization process, we mixed the support and lipase in different temperature, respectively. We found that there was an improvement in RML attaching with appropriate increasing temperature (Fig. 4B). The lipase solution with the initial concentration of 10, 20, 40, 80 mg/L was prepared. Though the relative immobilization efficiency decreased as the ratio of the enzyme increased, the total amount of immobilized RML further increased with a higher concentration of RML (Fig. 4C).

**Scheme 2:**
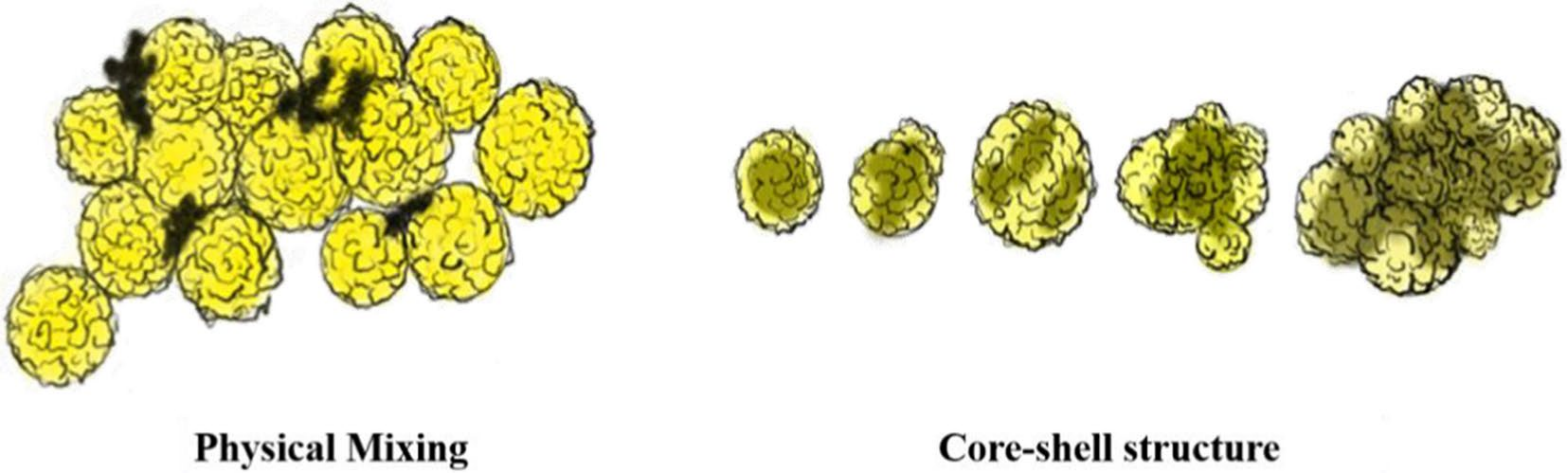
The scheme of two magnetic composites by different strategies: physical mixing and core–shell structure

After the immobilization, the hydrolysis of p-NPA (see details in Support information) was adapted to examine whether the immobilized RML is active and its stability. In this work, we employed the core–shell magnetic COFs (Fe_3_O_4_@COF-OMe) to enhance the recovery efficiency. Here, we also compared this strategy with the common mixing method. This tactic is to make COFs magnetic by mixing COFs and magnetic Fe_3_O_4_ nanoparticles in solutions (Fe_3_O_4_-COF-OMe), where the Fe_3_O_4_ nanoparticles are attached on the surface of COFs, shown in Scheme 2. The SEM & EDS mapping images display these two magnetic strategies in detail (Additional file 1: Figs. S12 and S13).

**Fig. 5:**
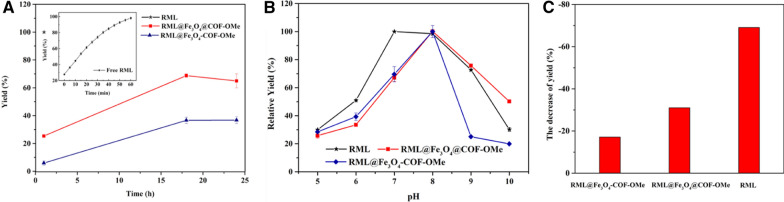
**A** Catalytic activity, **B** pH effect to and **C** thermal stability of free and immobilized RML (60 °C for 12 h ahead of test), examined by the hydrolysis of p-NPA

As shown in Fig. 5. After immobilization, there was a shuttle decrease in activity in hydrolysis of p-NPA of both immobilized RML (Fig. 5A). The best outcome could recover to 60% of the free enzyme (Fe_3_O_4_@COF-OMe) as the time prolonged. The Fe_3_O_4_@COF-OMe also showed good thermal and pH stability. The stability of the activity for both free RML and immobilized enzyme in different pH ranging from 5.0 to 10.0 was studied and is plotted in Fig. 5B. The result showed that the optimal pH altered slightly, from about 7.0 to 8.0. Thermal stability was investigated, which the biocomposites were stored at 60 ℃ for 12 h ahead of tests. It was observed that there is a decrease in activity for all of them, but the range of decrease was not significant for Fe-COFs immobilized RML (Fig. 5C). We found that although the Fe_3_O_4_-COF-OMe has a higher RML uptake, the enzyme activity of it did not perform well. It is due to the non-uniform and solid two-phase framework prepared by physical mixing and adhesion strategy, which was not enough to maintain the RML activity.

Based on the outcomes, Fe_3_O_4_@COF-OMe with better thermal stability and activity can indeed be optimal support for the subsequent study of transesterification reaction.

### Activity assay of transesterification reaction

**Fig. 6:**
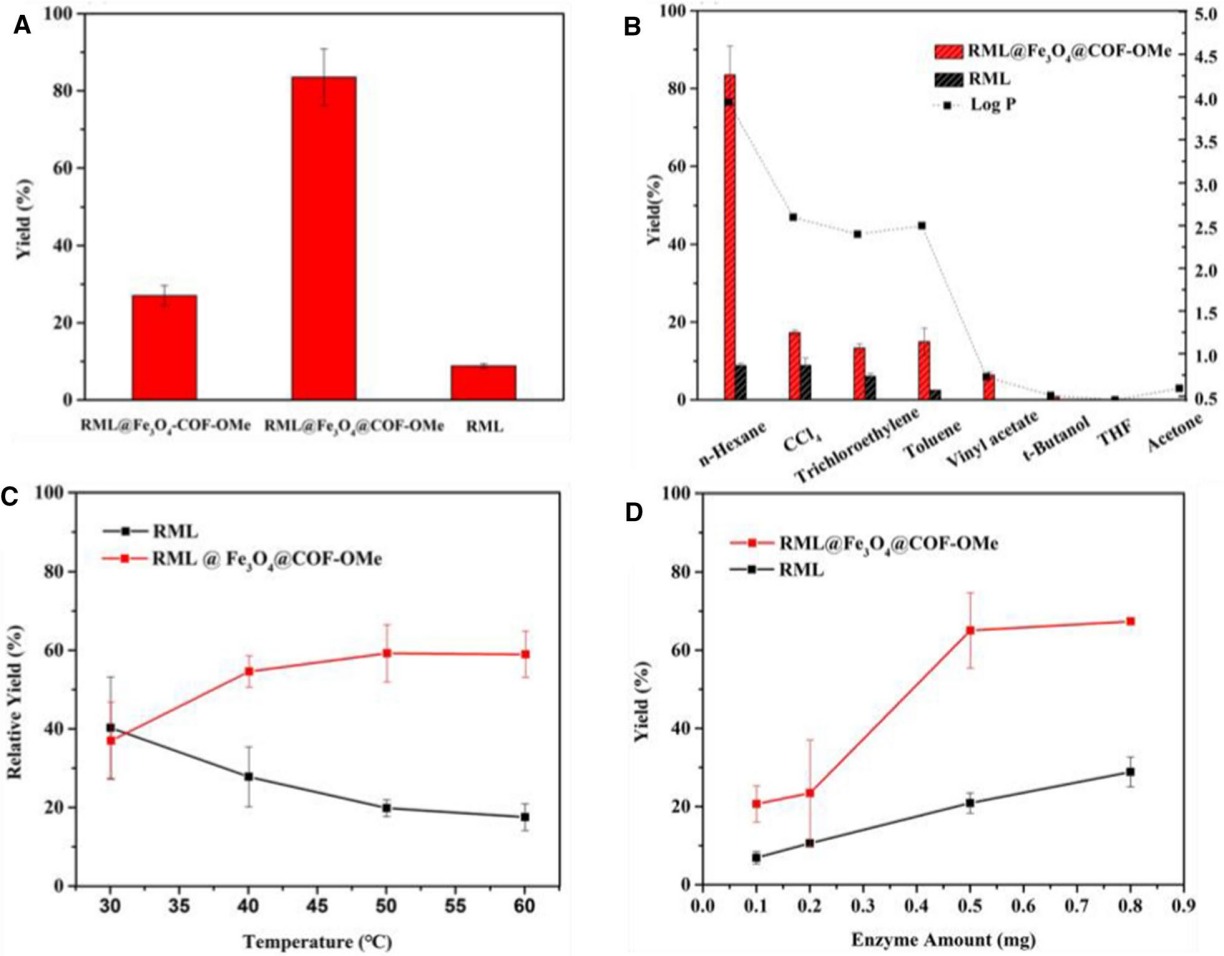
**A** Yields catalyzed by immobilized enzymes and free RML. The effect of **B** different solvents. **C** Temperature. **D** Different dosage of enzymes in transesterification reaction (equal RML amount for both free lipase and immobilized RML)

The activities of both free lipase and immobilized RML were determined by a transesterification reaction between 2-phenyl ethanol and vinyl acetate (Additional file 1: Figure S14). First of all, both Fe_3_O_4_@COF-OMe and Fe_3_O_4_-COF-OMe were adapt to catalyze under the same conditions. We found that this result was consistent with it of hydrolysis of p-NPA (Fig. 5), where it was Fe_3_O_4_@COF-OMe that performed better than Fe_3_O_4_-COF-OMe (Fig. 6A). At the same time, in the transesterification reaction, the yields of both immobilized enzymes were higher than that of free enzymes, which proved the excellent protective effect of carriers on the enzyme.

The solvent effect of the reaction in which n-Hexane functioned as a solvent is shown in Fig. 6B, with the highest yield up to 80%. Interestingly, according to the results, we found that the yields altered by the trends of the polarity of different solvents. In detail, the more hydrophobic the solvent was, the higher yields we got. As for the optimized solvent, whose log P value was largest, the yield was much higher than the others at the same time. Carbon tetrachloride, trichloroethylene, and toluene, whose polarity was similar, had almost the same yields of 20%. However, if the solvent was hydrophile, such as THF, acetone, the transesterification didn’t happen in it. To furtherly verify the hydrophobic solvents were conducive to this reaction, several homologous liquids of n-Hexane were adapted (Additional file 1: Fig. S15). n-Hexane, c-Hexane, and n-Heptane had similar yields, which indicated the hydrophobic solvents were beneficial in this work.

Then we investigated the influence of temperature on the reaction (Fig. 6C). Immobilized RML did better in the transesterification than the free enzyme. With the rise of temperature, the yield of the immobilized enzyme increased gradually and reached a peak at about 50 °C, while of liquid enzyme decreased continuously. The higher optimal temperature and activity could be related to a higher rigidity of the enzyme after immobilization, which could maintain high catalytic activity percentages under more drastic conditions [46–48].

The dosage of RML has also played an important role in a transesterification reaction, where excessive enzyme not only causes waste but also reduces the rate due to the aggregation. So here, we studied the yields of the reaction with different amounts of RML. As we can see in Fig. 6D, the yield of free RML still went up along with the increase of amount. For Immobilized RML, the yield didn’t show a significant rise when the RML on carrier changed to 0.8 mg. Considering the efficiency and economy of this reaction, 0.5 mg RML was used in every single sample assay. At the optimal conditions, n-Hexane as a solvent, the transesterification yield can reach about 80% with 0.5 mg of immobilized RML at 50 °C.

**Fig. 7:**
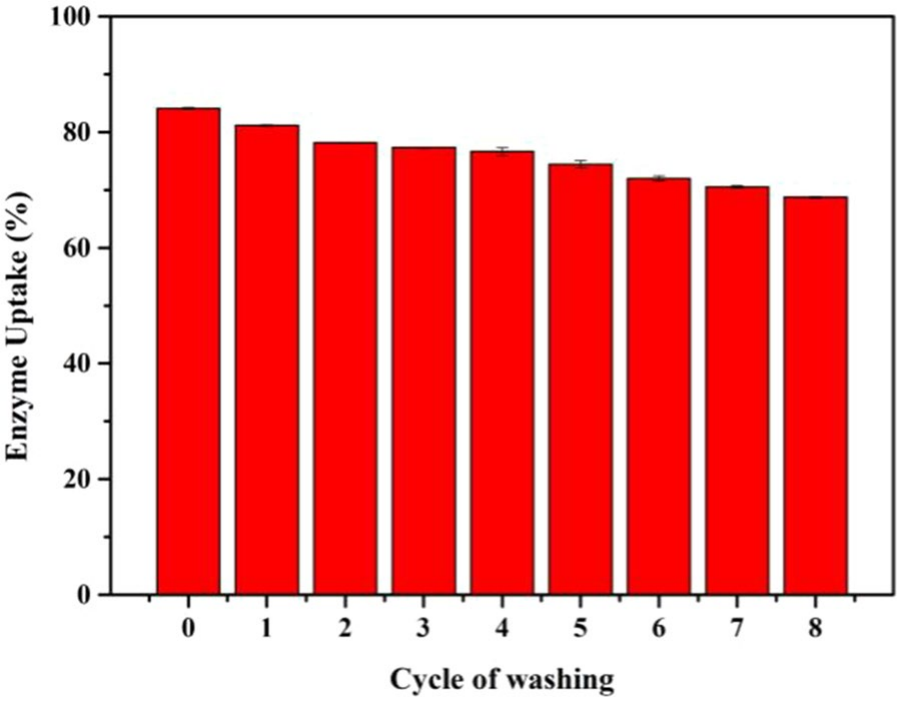
Leakage of the enzyme by washing operations in different cycles (Cycle 0: the origin immobilization uptake)

The preservation of activity by the protection of support in organic solvents and high temperature were shown above, where the immobilized RML always did better in different organic solvents and at over 30 °C than free lipase. To furtherly assess the function of COFs in protecting the catalytic ability of RML, the tolerance of immobilized RML against ultrasonic operation was investigated. As shown in Additional file 1: Figure S16, the yields of immobilized RML did not change significantly after ultrasonic treatment, and always higher than that of free RML at the same time. Here we also studied the leakage ratio of RML by washing the immobilized lipase (Fig. 7). As we can see, the amount of loss of RML for every single wash was about 2% and the total leakage ratio was less than one-fifth after the 8 wash cycle, which indicated a good ability to preserve the lipase from washing operation.

### Production of biodiesel

**Fig. 8:**
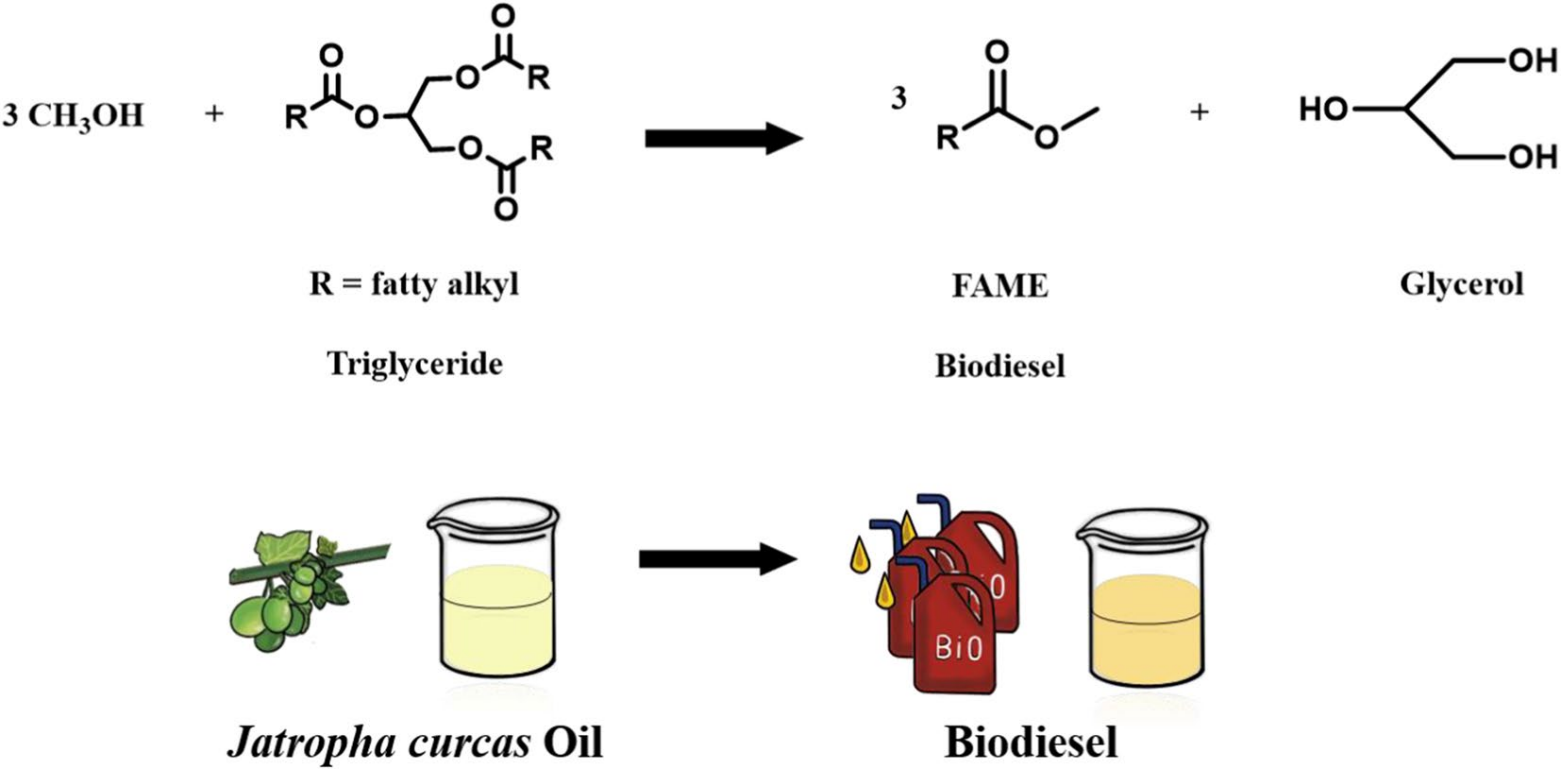
Schematic representation of the production of biodiesel

Having established the efficiency of RML@Fe_3_O_4_@COF-OMe in the transesterification reaction of 2-phenol ethanol and vinyl acetate, then we studied its catalytic ability in the production of biodiesel from inedible *Jatropha curcas* Oil (Table 1). The outcomes^a^ catalyzed by immobilized RML are much better than those by free RML, with a yield of 67.8% and 5.1%, respectively. It is noticed that there is an obvious loss in enzymatic activity when the amount of methanol exceeds the stoichiometric ratio (3:1). This is due to the inhibitory effect of methanol, and the activity is irreversibly inactivated [35]. Compared to free RML, magnetic Fe_3_O_4_ nanoparticles could protect RML from the methanol, as the product could be detected with a satisfactory yield. It is noticed that the protective effect is not permanent although the activity could be maintained if the concentration of methanol is doubled (Entry 4, Table 1). However, if the amount of methanol is excessive too much (15:1 and 30:1), there is a huge loss in yield. In a word, the nanoparticle efficiently improved the stability and maintained the activity of the enzyme in practical application (Fig. 8).

**Table 1:** RML@Fe_3_O_4_@COF-OMe-catalyzed production of biodiesel by *Jatropha curcas* oil^a^

Entry	Enzyme	Methanol (μL)	Yield (%)
1	Free RML	10	5.1
2	Free RML	20	Trace
3	RML@Fe_3_O_4_@COF-OMe	10	67.8
4	RML@Fe_3_O_4_@COF-OMe	20	72.3
5	RML@Fe_3_O_4_@COF-OMe	50	Trace
6	RML@Fe_3_O_4_@COF-OMe	100	Trace

^a^Reaction conditions: *Jatropha curcas* Oil (0.15 mmol), methanol (0.45 mmol, 10 μL), n-Hexane (3 mL), RML@Fe_3_O_4_@COF-OMe/RML (5 mg of RML), and 50 °C at 100 rpm for 48 h. The yields were determined by GC

## Conclusion

In summary, we successfully prepared a core–shell magnetic COFs structure (Fe_3_O_4_@COF-OMe) in a facile way. The resultant magnetic COFs exhibited a great uptake of RML and facilitated the separation taking advantage of features of high surface area, porosity, good chemical stability, and strong magnetic response. The support could maintain the activity of RML and perform well in practical applications. We applied the immobilized RML into the production of biodiesel from un-editable *Jatropha curcas* Oil and obtained a satisfactory yield. This first successful attempt of COFs-based immobilized lipase demonstrates their promising applications in the production of biodiesel.

## Materials and methods

### Materials

All chemicals and reagents were commercially available and used without further purification. 2,5-dimethoxyterephthalaldehyde (DMTP), 1,3,5-tris(4-aminophenyl)-benzene (TPB) were purchased from Shanghai KaiYuLin pharmaceutical technology co., LTD. *Rhizomucor miehei* lipase (RML) was supplied by Sigma-Aldrich. Iron(II) chloride tetrahydrate (FeCl_2_·4H_2_O), Iron chloride hexahydrate (FeCl_3_·6H_2_O) were obtained by Chengdu Hua Xia Chemical Reagents Co., LTD. Rhodamine B isothiocyanate (RBITC), 4-Nitrophenyl acetate (p-NPA) were purchased by Aladdin Chemistry Co., Ltd (Shanghai, China). Methanol (HPLC), Tetrahydrofuran (anhydrous, HPLC) were supplied by Thermo Fisher Scientific (China) Co., LTD. Bradford Protein Assay Kit was obtained by Beyotime Biotech Inc, Shanghai, China. The other chemical reagents and solvents were purchased by Chengdu Chron Chemicals. Co., LTD. *Jatropha curcas* Oil was bought from market Liangzhou, Sichuan province.

### Characterization

Powder X-ray diffraction (PXRD) data were collected on X’Pert Pro MPD DY129 X-ray diffractometer (40 kV, 40 mA) using Cu Kα (*λ* = 1.5406 Å) radiation. The gas adsorption isotherms were collected on a surface area analyzer, Quantachrome Instruments version 3.01 Autosorb Station 2. The N_2_ sorption isotherms were measured at 77 K using a liquid N_2_ bath. Scanning electron microscopy (SEM) images were collected using a JSM-5900LV (JEOL, Japan). Transmission electron microscopy (TEM) images were collected using a HITACHI H-600, operating at 75 kV. IR spectra were recorded on a Nicolet Impact 410 FTIR spectrometer. The confocal laser scanning microscopy (CLSM) data were collected on a Leica SP5 under an excitation *λ*_ex_ = 488 nm and *λ*_ex_ = 543 nm. The vibrating sample magnetometer (VSM) data were measured by LakeShore7404. Thermal Gravity analysis (TG/TGA) was tested and analyzed by METTLER TOLEDO TGA/DSC2/1600.

### ***Preparation of magnetic Fe***_***3***_***O***_***4***_*** nanoparticles***

The magnetic Fe_3_O_4_ was prepared by a facile coprecipitation method. First, FeCl_2_·4H_2_O (0.74 g, 3.7 mmol) and FeCl_3_·6H_2_O (1.22 g, 7.5 mmol) were dissolved in deionized water (25 mL) by vigorous stirring. Then, 30% (w/v) NaOH solutions (7 mL) was added dropwise, keeping stirring for another 1–2 h. The obtained black magnetic precipitate was washed by deionized water several times until the pH values decrease to 7.0. With the help of the external magnetic field, the nanoparticles were separated and dried under vacuum.

### Preparation of core–shell magnetic COFs (Fe_***3***_***O***_***4***_@COF-OMe)

The preparation of magnetic COFs was similar to the preparation of COF-OMe, addition the magnetic Fe_3_O_4_ particles along with monomers, The COF-OMe shell is shaped and formed core–shell structure. In brief, to a 50 mL of acetonitrile solution containing DMTP (0.24 mmol) and TPB (0.16 mmol), Fe_3_O_4_ nanoparticles (30 mg) was added. After sonicated for 5 min. acetic acid (17.5 M, 3 mL) was dropped into the suspension. The reaction proceeded at room temperature for 2 h. The grey–yellow precipitates can be separated by the external help of a magnet. After washed by methanol 3 times, the remaining monomers were cleared from reaction mixtures by anhydrous tetrahydrofuran using Soxhlet extraction for 2 days. The product was dried under vacuum at 50 ℃ for 24 h to afford Fe_3_O_4_@COF-OMe.

### Preparation of RBITC-labelled RML (RBITC-RML).

RBITC (0.5 mg) and RML solution (3 mL) were added into 4 mL of PBS buffer solution (pH 7.4) and left in darkness overnight under gentle stirring. The RBITC-tagged RML (RBITC-RML) was obtained by dialysis for 2 days in PBS buffer.

### Immobilization capacity of RML

The RML was immobilized by physical adsorption in PBS buffer. The support material was first immersed into PBS buffer. 80 mg/L of RML was then added. Keep the mixture in a rolling incubator for 8 h at 20 rpm at room temperature. The composite and supernatant were collected by centrifugation and magnet to observe the activity and immobilization efficiency. The uptake capacity was measured by the method of Coomassie blue staining with the use of Bradford Protein Assay Kit (see details in Support Information). The enzyme uptake capacity is calculated by:

$$ {\text{Ca}}\, = \,\left( {C_{0}  - C} \right){\text{ }}V/M_{{\text{s}}} $$

*C*_0_ represents the initial concentration of RML solutions, while *C* is the concentration of supernatant after immobilization. *V* is the total volume of the system. M_s_ is the mass of support.

Enzymatic activity after immobilization process was measured through the hydrolysis of p-NPA. To a tube, p-NPA (500 μL, 2 μmol/mL) and PBS buffer (2 mL, pH = 7.4) with immobilized RML (or 2 mL total volume of free enzyme and PBS buffer solution) were added. The absorbance at 405 nm was detected 1 h later. Finally, the product concentrations were corrected for the auto-hydrolysis of p-NPA and also the absorbance of p-NPA left in the solution. (See details in Support Information). Ahead of thermal stability tests, both free RML and Immobilized RML were stored at 60 °C for 12 h.

### Activity assay for transesterification reaction.

The transesterification of 2-phenylethanol and vinyl acetate were chosen to determine the activity of immobilized RML in production of biodiesel. In general, 2-phenylethanol (20 μL) and vinyl acetate (40 μL) were added into a tube with 2 mL of solvent, 150 rpm for 24 h. Different bio-complex, solvent, temperature, and amount of RML in carrier were optimized. The yields of transesterification were determined by HPLC analysis with a Waters-HPLC on C18 columns using methanol/water (70:30) as the eluent.

### Production of biodiesel

In the experiment of production of Biodiesel, to a tube of 3 mL of the solvent containing *Jatropha curcas* Oil (0.15 mmol) and methanol (0.45 mmol), immobilized RML was mixed. Then, the system was put into a shaker at 150 rpm for 48 h at conditions optimized by transesterification of 2-phenylethanol and vinyl acetate. The yields were determined by GC-analysis.

## Supplementary Information

**Additional file 1**. Detail experimental procedures, materials, PXRD, SEM, etc. (EIS).

## Data Availability

The data supporting the results of the article are included in this manuscript and supplementary information.

## Abbreviations

COFs: Covalent-organic frameworks
MOFs: Metal–organic frameworks
RML: *Rhizomucor miehei* Lipase
PXRD: Powder X-ray diffraction
BET: Brunauer–Emmett–Teller
FTIR: Fourier transform infrared
RT: Room temperature
Fe_3_O_4_@COF-OMe: COF-OMe-coated Fe_3_O_4_ nanoparticles to form core–shell structure.
Fe3O4-COF-OMe: Physically mixtures
GC: Gas chromatography
SEM: Scanning electron microscopy
TG(TGA): Thermal gravity analysis
TEM: Transmission electron microscopy
CLSM: Confocal laser scanning microscopy
VSM: Vibrating sample magnetometer
HPLC: High performance liquid chromatography
RBITC: Rhodamine B isothiocyanate
FITC: Fluorescein isothiocyanate
PBS: Phosphate buffer saline
p-NPA: 4-Nitrophenyl acetate
