# Supplementary material for: Magnetic COFs as satisfactory support for lipase immobilization and recovery to effectively achieve the production of biodiesel by maintenance of enzyme activity
Source: Biotechnol Biofuels. 2021 Jul 14;14:156. doi: 10.1186/s13068-021-02001-0 (PMC8278614; doi:10.1186/s13068-021-02001-0)
Supplement: Supplementary file 1 — Additional file 1. Detail experimental procedures, materials, PXRD, SEM, etc. (EIS). [file 13068_2021_2001_MOESM1_ESM.docx]

**Supporting Information**

**Magnetic COFs as satisfied support for lipase immobilization and recovery to effectively achieve the production of biodiesel by great maintenance of enzyme activity**

*Zi-Wen Zhou**^a^, Chun-Xian Cai^a^, Xiu Xing^a^, Jun Li^a^, Zu-E Hu^a^, Zong-Bo Xie^b^, Na Wang ^a^*, Xiao-Qi Yu ^a^**

*^a^Key Laboratory of Green Chemistry and Technology (Ministry of Education), College of Chemistry, Sichuan University, Chengdu 610064, P. R. China.*

*E-mail: wnchem@scu.edu.cn; xqyu@scu.edu.cn; Fax: +86-28-85415886; Tel: +86-28-85415886*

*^b^Jiangxi Province Key Laboratory of Synthetic Chemistry, School of Chemistry, Biology and Material Science, East China University of Technology, Nanchang 330013, P. R. China.*

*E-mail:* *zbxie@ecut.edu.cn.*

**Table of Contents**

1. **Experiment Section ……………………………………………………………………… 2**
   1. **Determination of loading capacity of RML by using BSA standard curves …….. 3**
   2. **Enzymatic activity assay ……………………………………………………………. 3**
   3. **Synthesis of COF-OMe …………………………………………………………... 5**
2. **Supplementary Figures and Tables ……………………………………………………... 5**

**1. Experiment Section**

**1.1 Determination of loading capacity of RML by using BSA standard curves.**

Preparation of BSA standard reagents:

The BSA standard solution was prepared by solving 10mg BSA in deionized water, diluting to 10ml. The concentration of acquired BSA standard solution was 1mg/mL.

Coomassie brilliant blue staining methods (Bradford Protein Assay Kit).

I: To a 96-hole plates, 200μL of G250 staining was added into every single holes.

II: Add 50μL of BSA standard solutions with concentration gradient (Table SA).

III: Incubate about 10min.

IV: Determine the Absorbance at 595nm.

V: Prepare BSA standard curves (Figure SA). Determine the unknown protein samples by this curve.

**
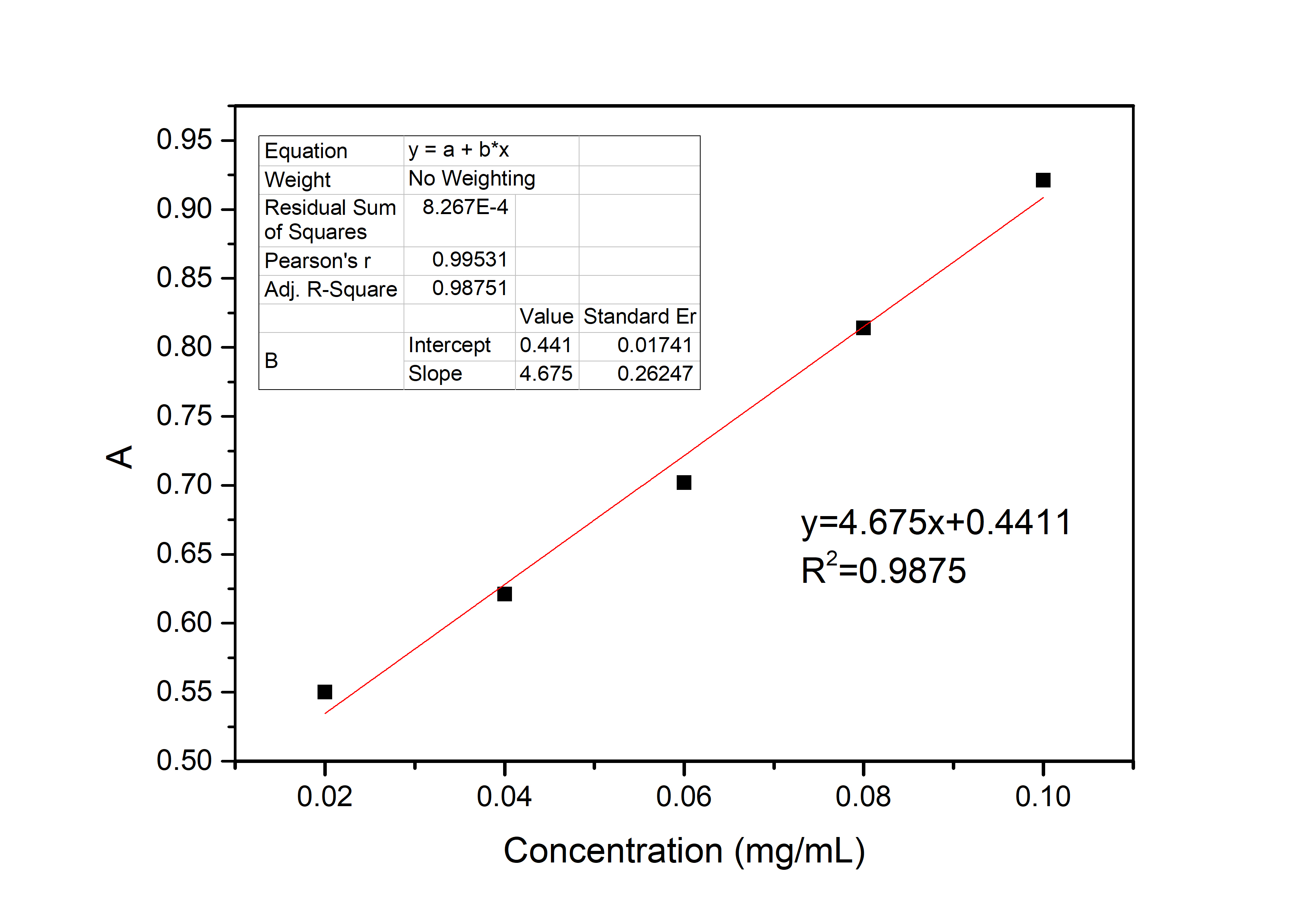
**

Figure SA: The BSA standard curves using Coomassie brilliant blue staining assays

| Entry | PBS buffer | BSA standard solutions | Concentration |
| --- | --- | --- | --- |
| 1 | 900μL | 100μL | 0.1mg/mL |
| 2 | 920μL | 80μL | 0.08mg/mL |
| 3 | 940μL | 60μL | 0.06mg/mL |
| 4 | 960μL | 40μL | 0.04mg/mL |
| 5 | 980μL | 20μL | 0.02mg/mL |

Table SA: The preparation of BSA standard solution with concentration gradient

**1.2 Enzymatic activity assay**

Determination of auto-hydrolysis of p-NPA:

We investigated the enzymatic activity of RML and immobilized RML by hydrolysis of p-NPA. It should be considered that p-NPA will hydrolyze automatically. So the problem that auto-hydrolysis of the substrate 4-nitrophenyl acetate had to be addressed. This was done by investigating the auto-hydrolysis of 0.4 μmol/mL of p-NPA of over time (Figure SB).


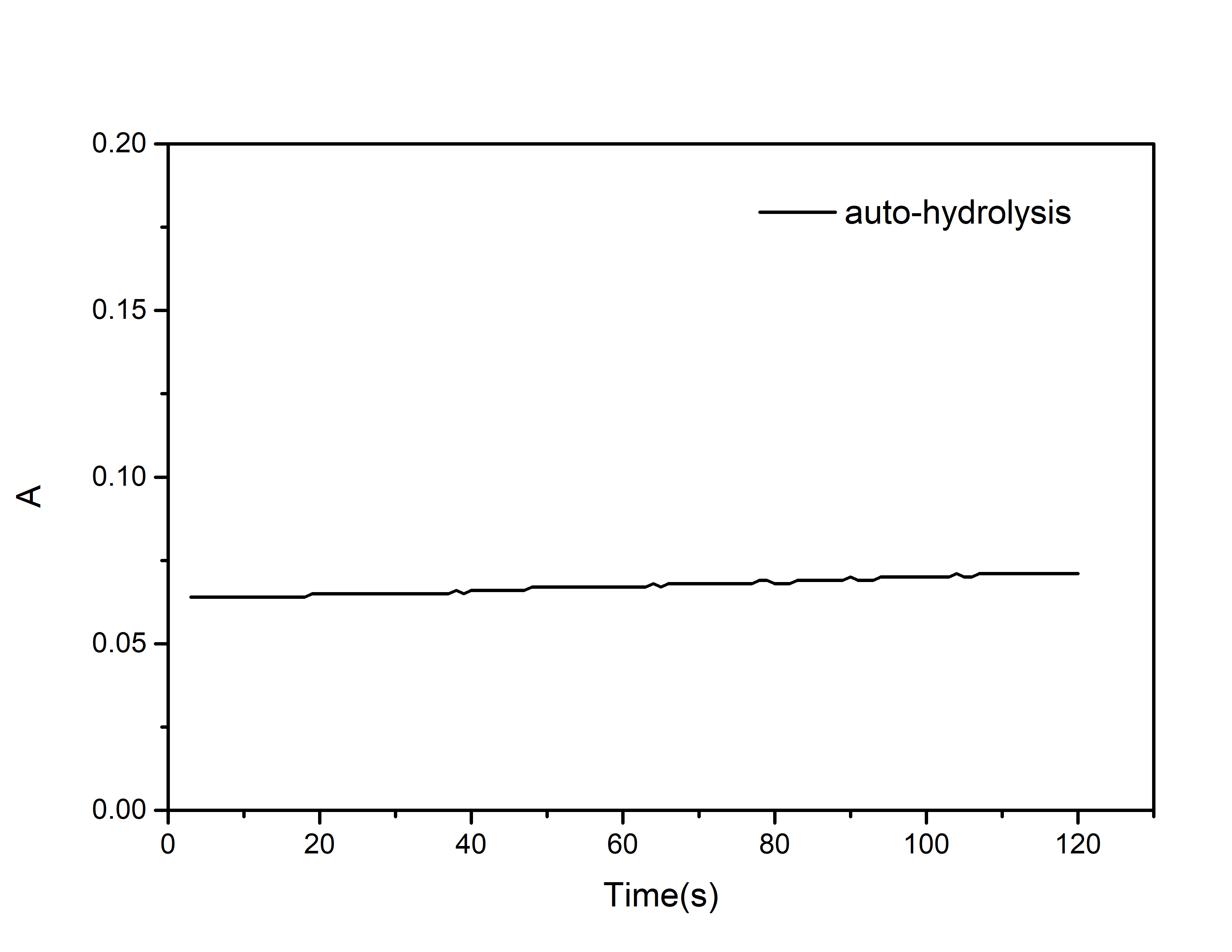


Figure SB: The auto-hydrolysis of p-NPA

Preparation of p-NP standard curves:

Refer to Table SB, the p-NP standard curve (Figure SC) was determined by preparing a series of p-NP standard solutions with concentration gradient and detecting their absorbance at 405nm.


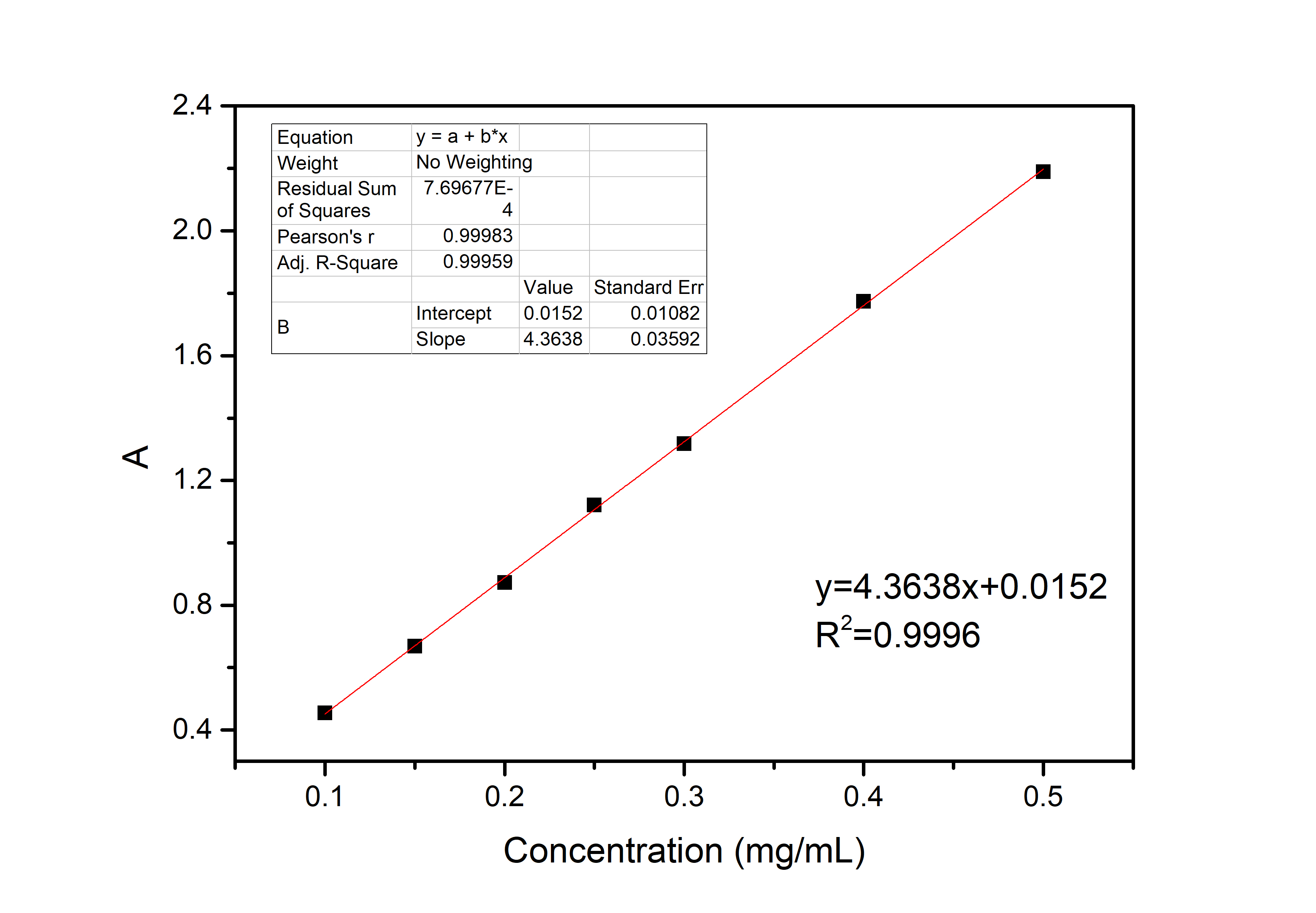


Figure SC: The standard curves of p-NP

Table SB The preparation of p-NP standard solution with concentration gradient

| Entry | p-NP | PBS buffer | Concentration |
| --- | --- | --- | --- |
| 1 | 500μL | 9500μL | 0.1 μmol/mL |
| 2 | 750μL | 9250μL | 0.15 μmol/mL |
| 3 | 1000μL | 9000μL | 0.2 μmol/mL |
| 4 | 1250μL | 8750μL | 0.25 μmol/mL |
| 5 | 1500μL | 8500μL | 0.3 μmol/mL |
| 6 | 2000μL | 8000μL | 0.4 μmol/mL |
| 7 | 2500μL | 7500μL | 0.5 μmol/mL |

The enzyme activity assay

To a tube, 500μL, 2μmol/mL of p-NPA and 2000μL of PBS buffer with immobilized RML (or 2000μL total volume of free enzyme and PBS buffer solution) were added into a tube. The absorbance at 405nm was detected. Finally, the product concentrations were corrected for the auto-hydrolysis of p-NPA and also the absorbance of p-NPA left in the solution.

**1.3 Synthesis of COF-OMe**

To a 50mL of acetonitrile solution containing DMTP (0.24 mmol) and TPB (0.16 mmol) were added. After sonicated for 5 min. acetic acid (17.5 M, 3 mL) was dropped into the suspension. The reaction proceeded at room temperature for 2 h. The yellow precipitate was obtained. After washed by methanol 3 times, the remaining monomers were cleared from reaction mixtures by anhydrous tetrahydrofuran using Soxhlet extraction for 2 days. The product was dried under vacuum at 50 ^o^C for 24 h to afford COF-OMe.

1. **Supplementary Figures and Tables**


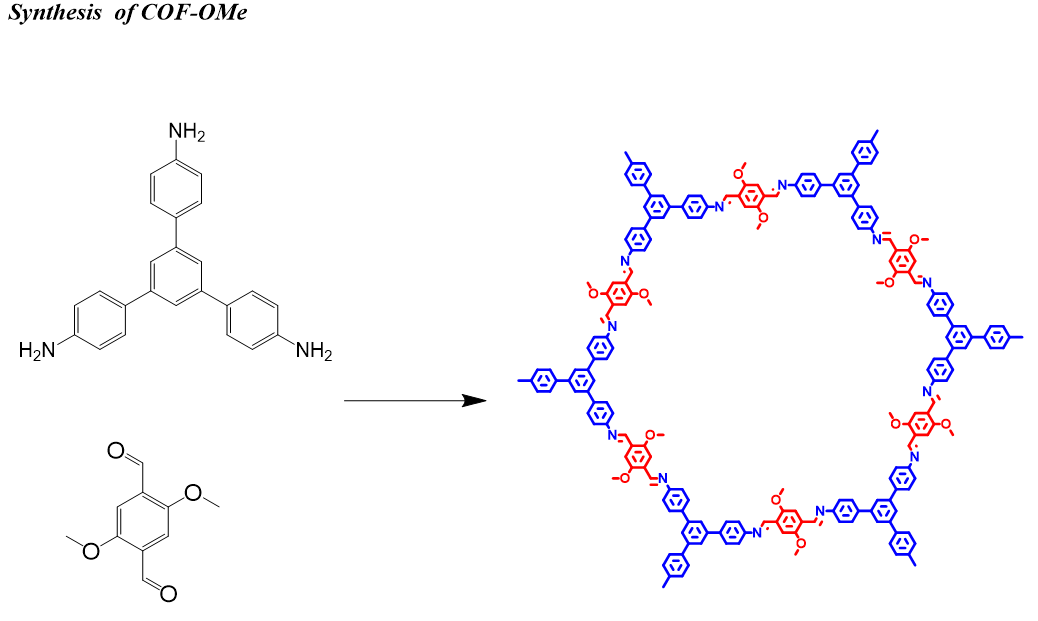


Figure S1. The synthesis of COF-OMe.


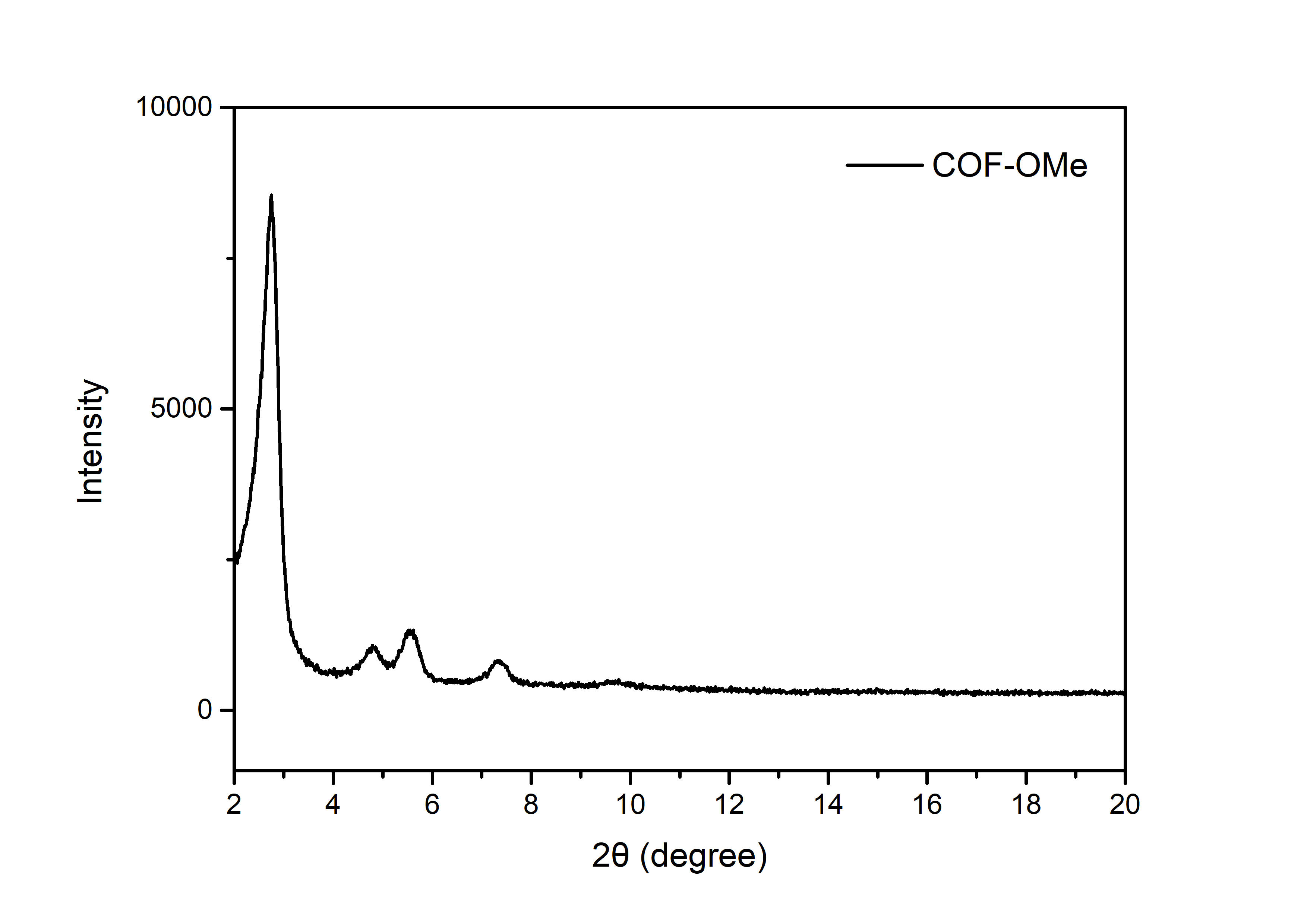


Figure S2. PXRD patterns of COF-OMe (prepared in acetonitrile)


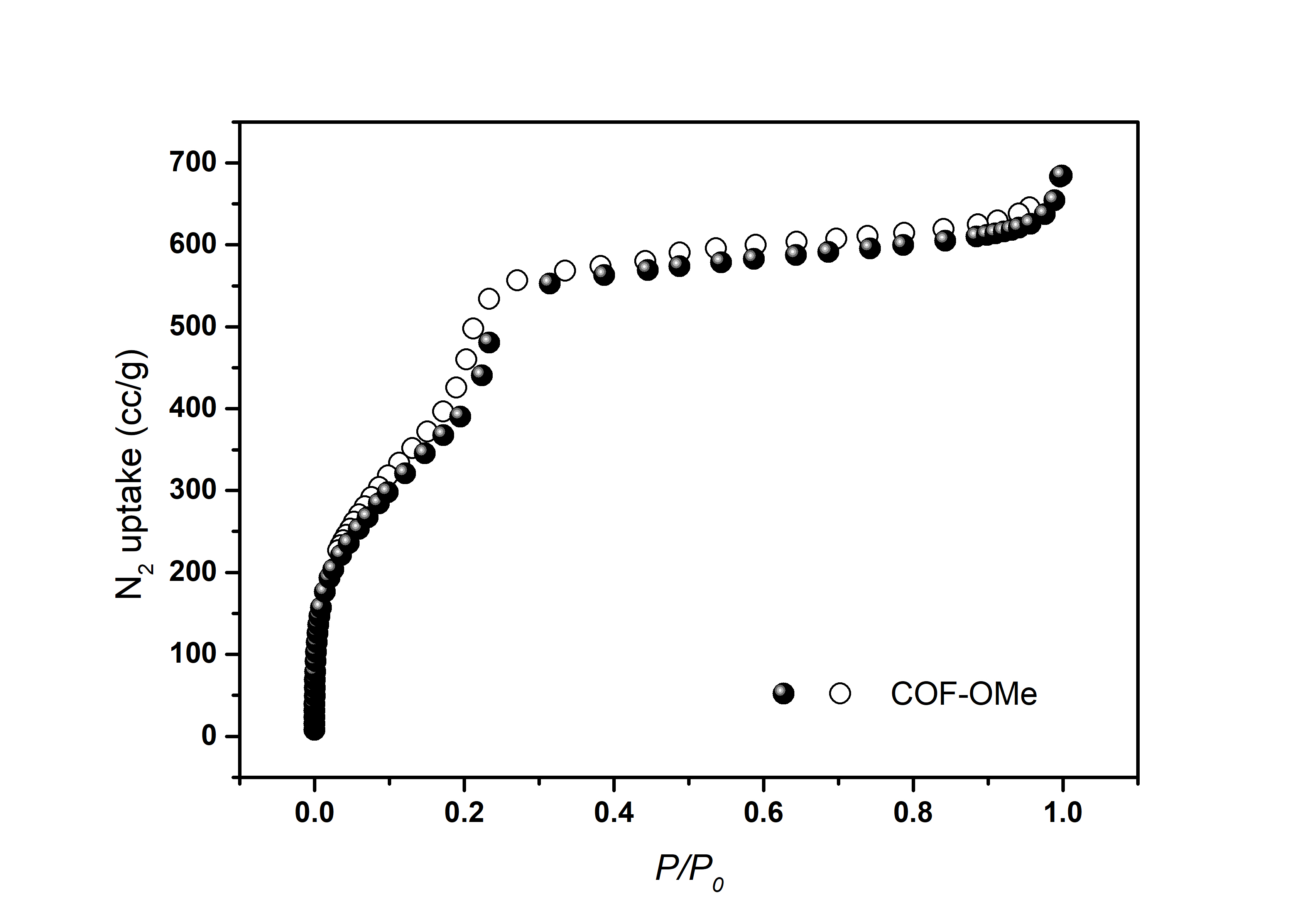


Figure S3. N_2_ sorption isotherms collected at 77 K.


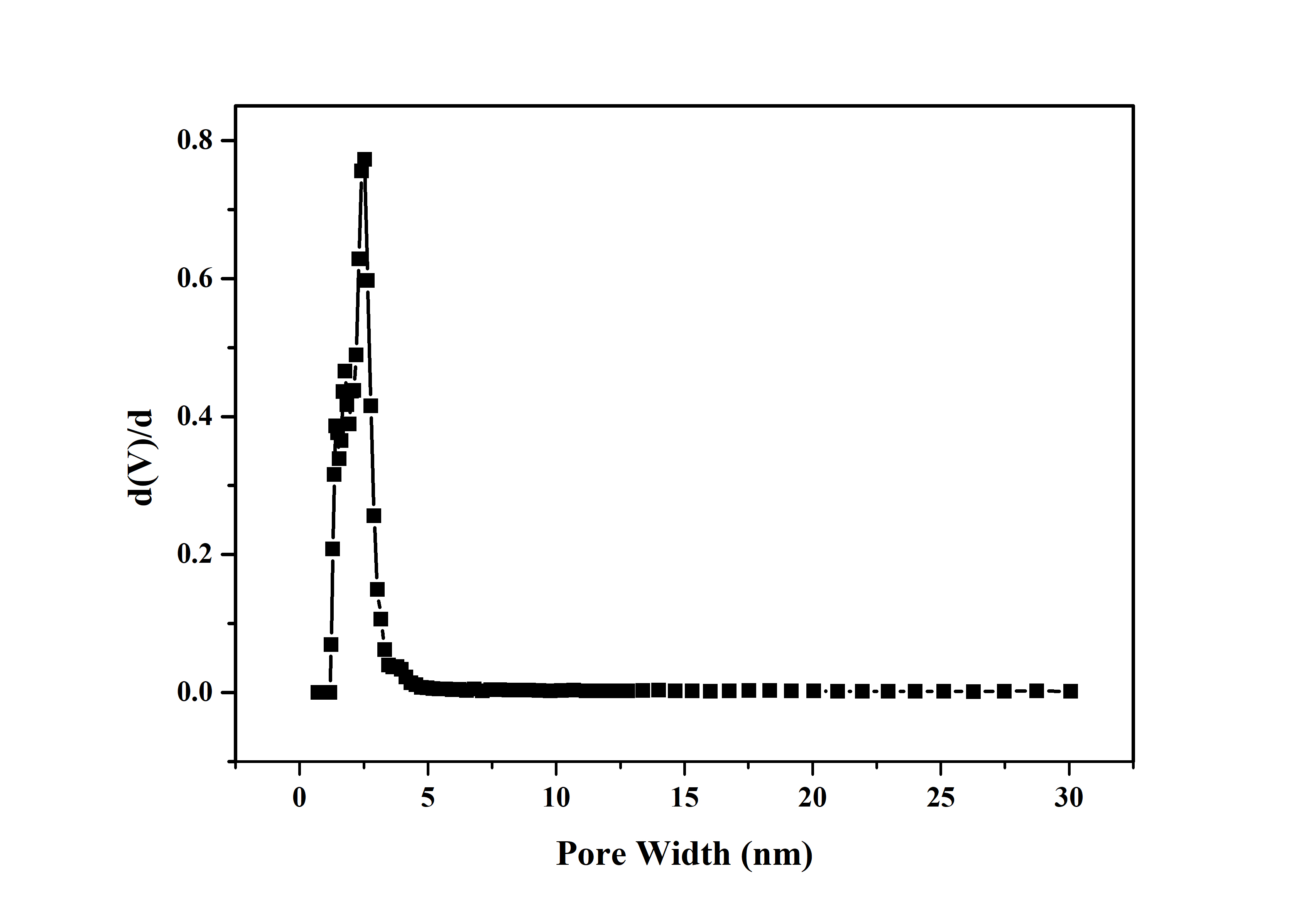


Figure S4. Pore size distribution based on the nonlocal density functional theory method.


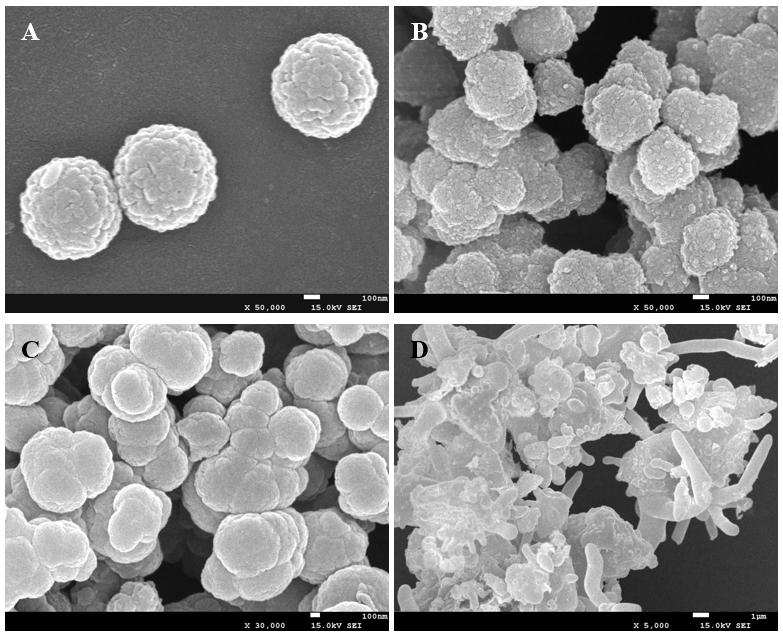


Figure S5. The SEM images of COF-OMe prepared by facile method in different solvents: A) Acetonitrile, B) DMSO, C) Toluene, D) n-Hexane.


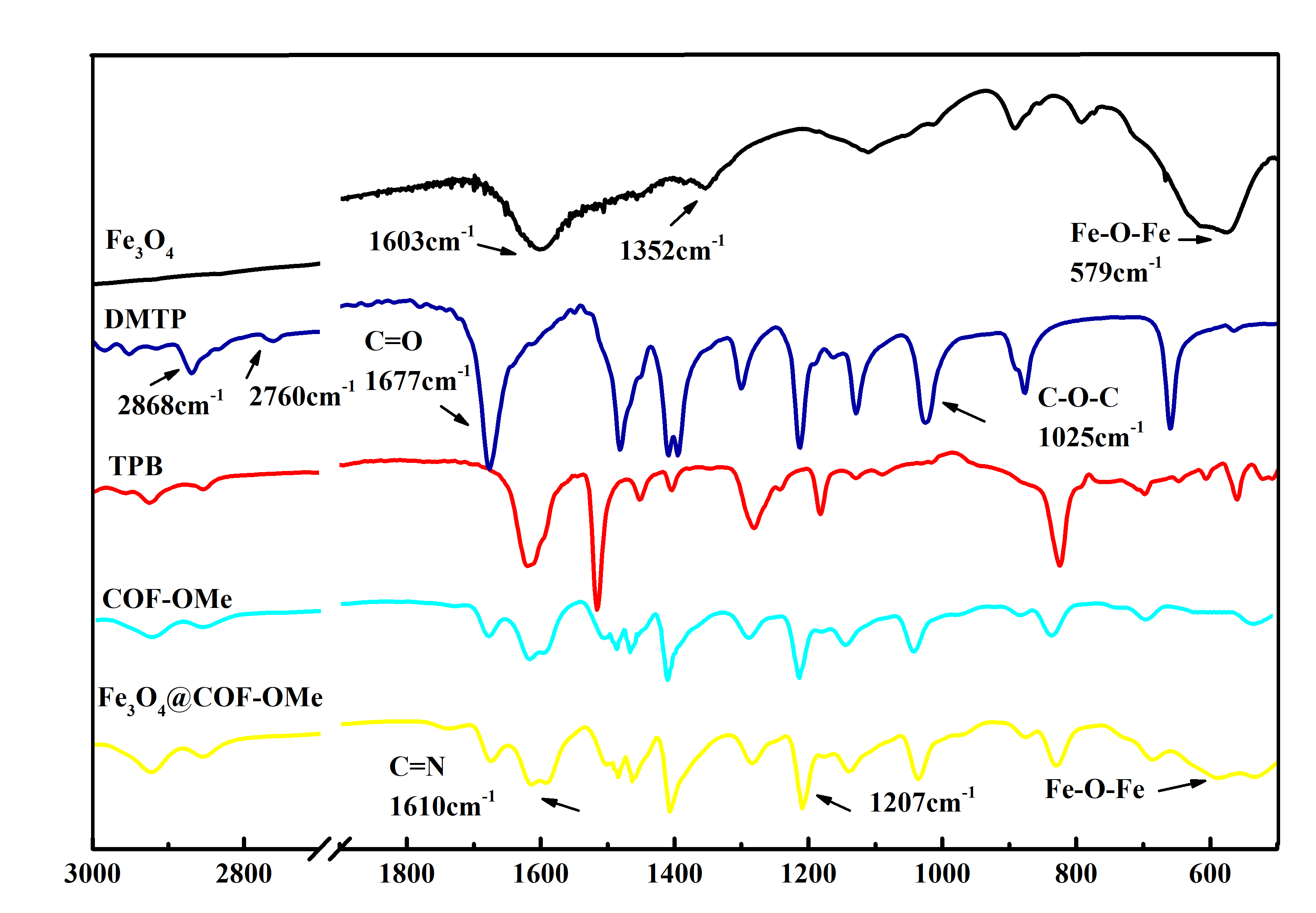
Figure S6. The FTIR spectrum of Fe_3_O_4_ (black), monomers of COF-OMe (DMTP for blue, TPB for red), COF-OMe (cyan) and Fe_3_O_4_@COF-OMe (yellow).

The FTIR spectrum of Fe_3_O_4_ contains a band at 579 cm^-1^, which is assigned to characteristic Fe-O-Fe stretch. The characteristic absorption bands of monomer 2,5-dimethoxyterephthalaldehyde (DMTP) at 2868 cm^-1^, 2760 cm^-1^ and 1677 cm^-1^ demonstrate the existence of aldehyde groups, where the 1677 cm^-1^ is assigned to the C=O stretching. The bands at 1207 cm^-1^, 1025 cm^-1^ attributed to the aromatic =C-O-C asymmetric and symmetric vibration respectively and the peak at 1610 cm^-1^ assigned to the C=N stretch mode are observed in the curve of Fe_3_O_4_@COF-OMe, which means the successful synthesis of COF-OMe by condensation of aldehydes and amines^1^.


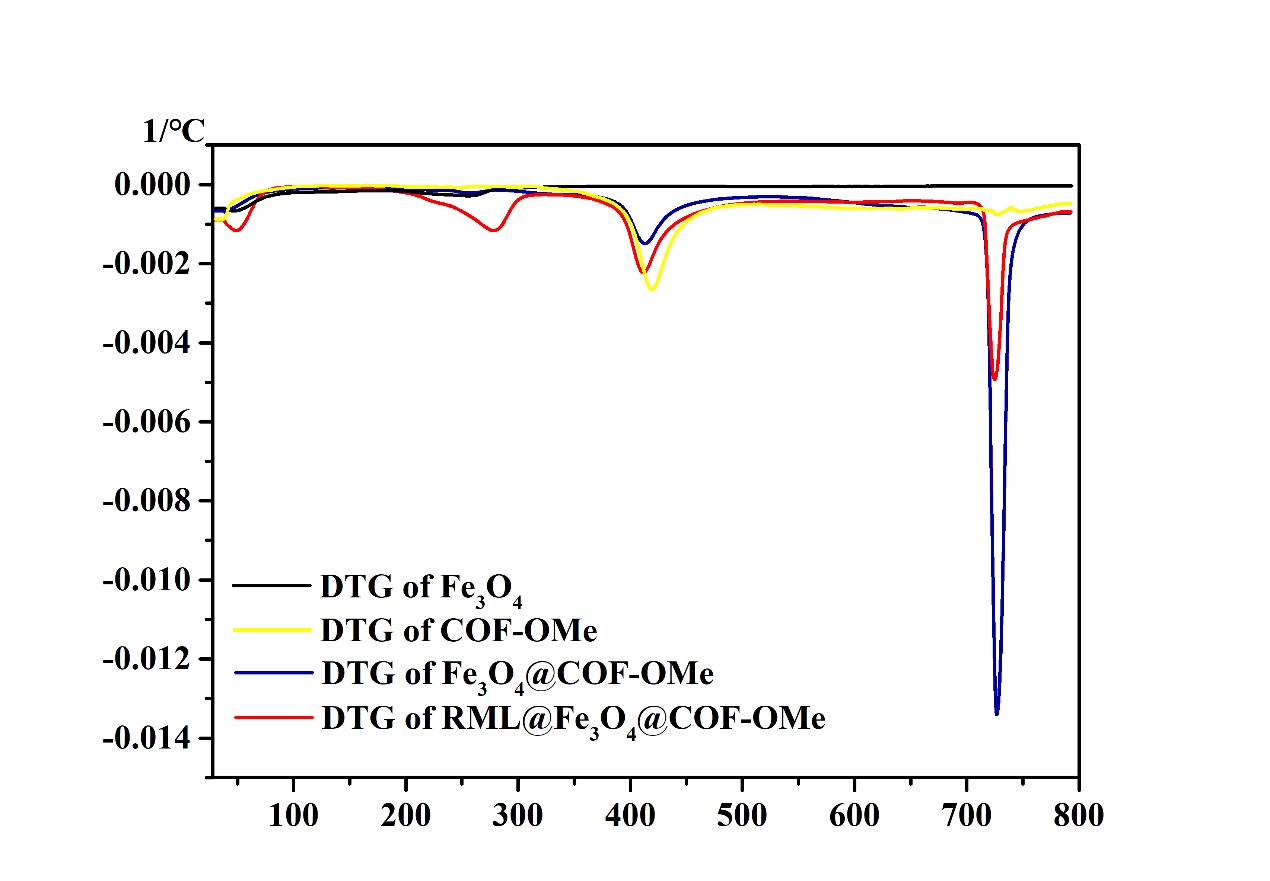
Figure S7. The differential thermal gravity (DTG) image

**(A)**


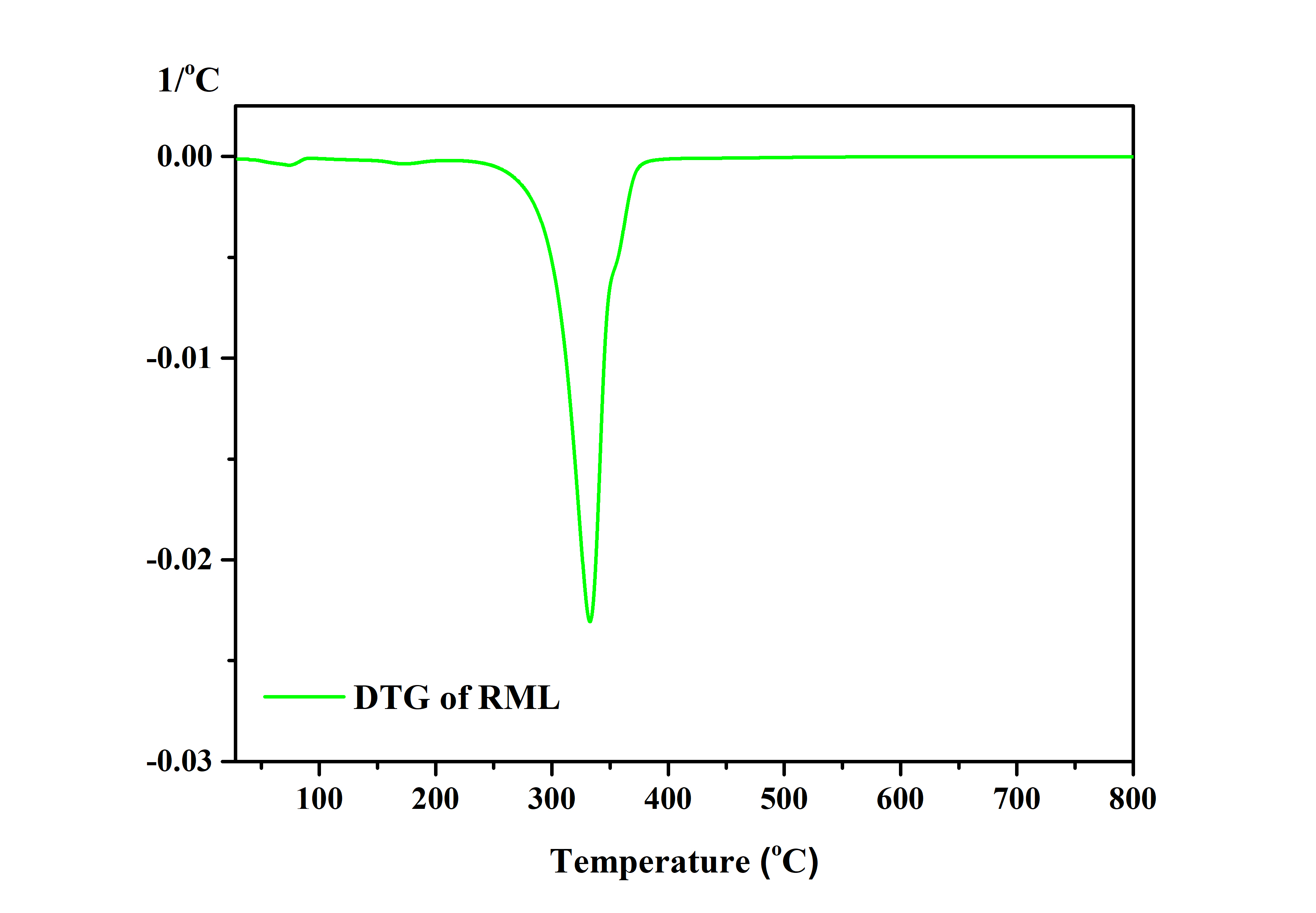


**(B)**


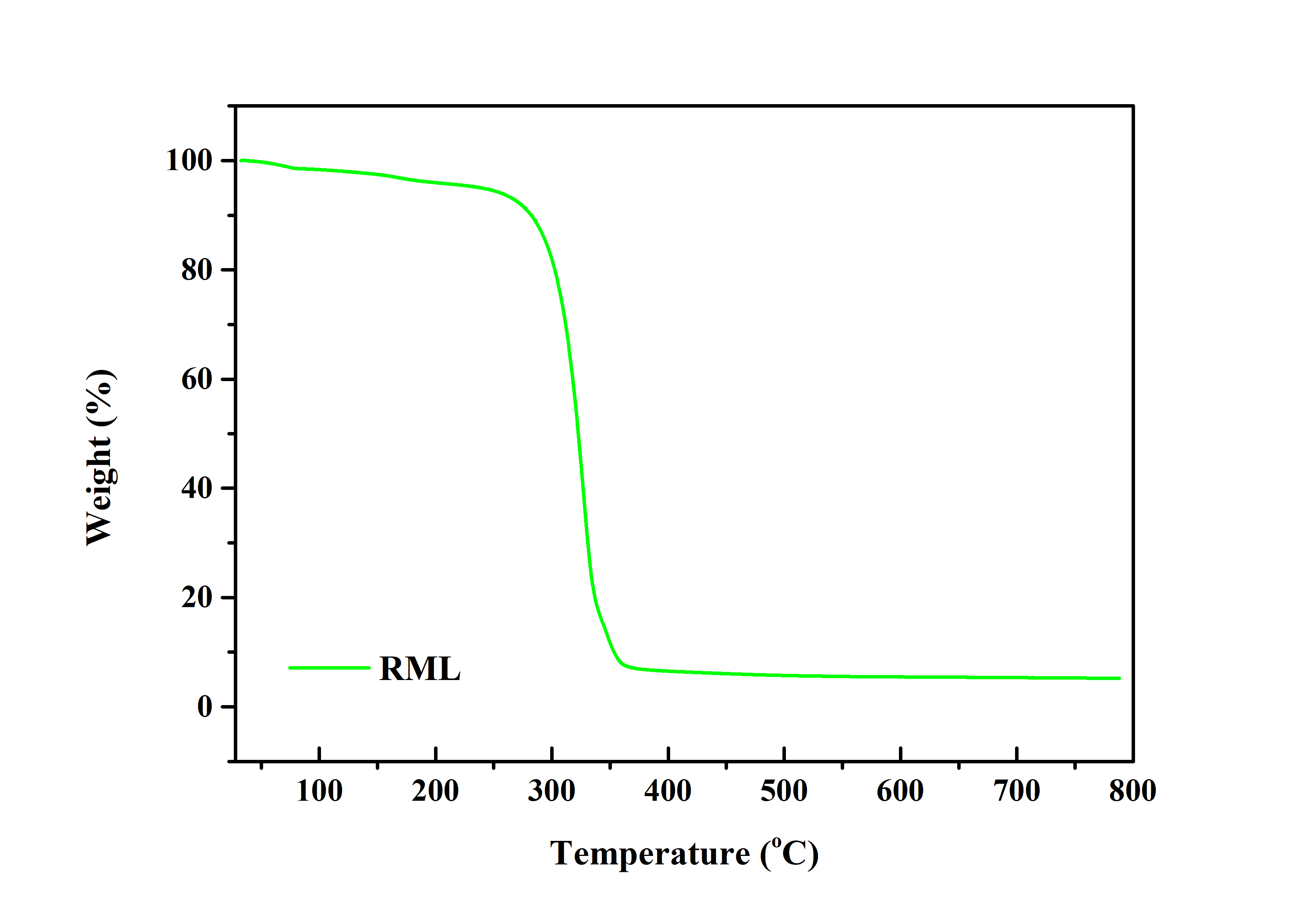
Figure S8. A) The TGA and B) DTG curve of Free RML.


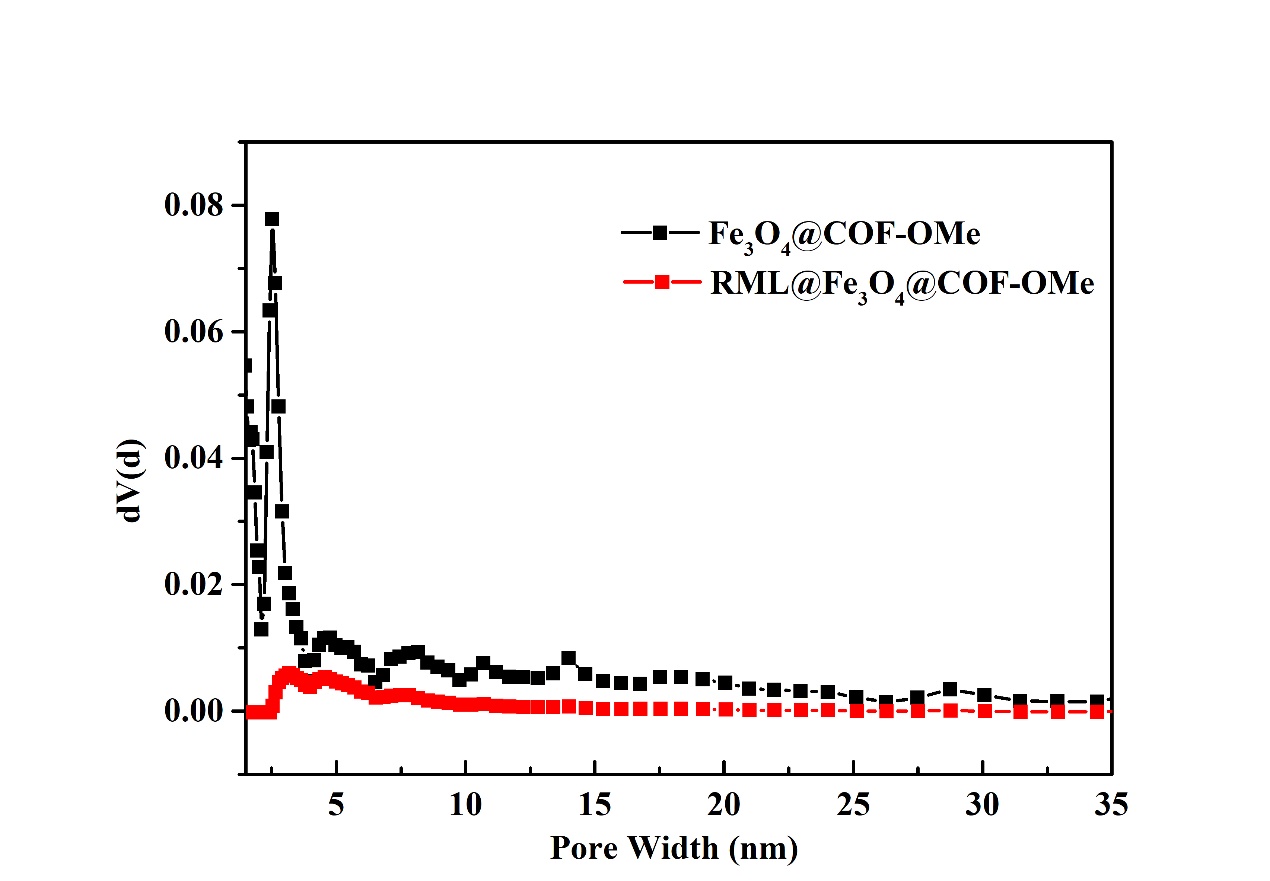
Figure S9. Pore distribution of Fe_3_O_4_@COF-OMe and RML@Fe_3_O_4_@COF-OMe


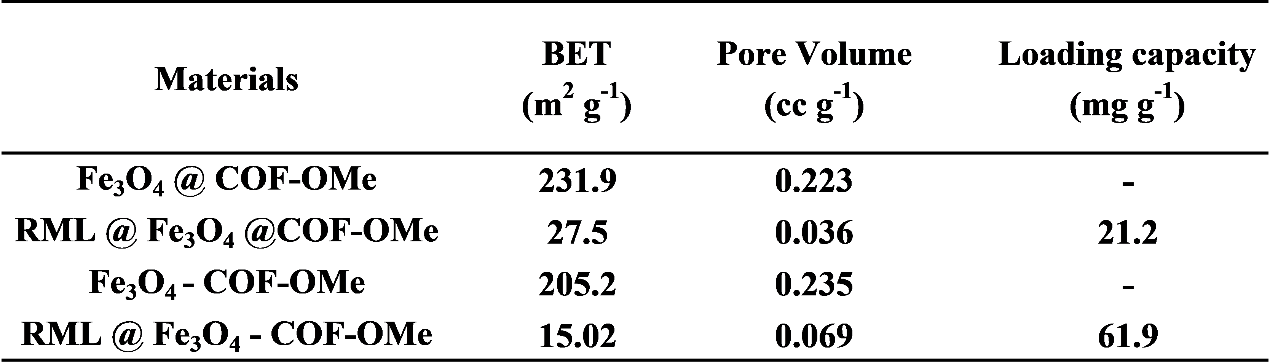
Table S1. The textural parameters of two magnetic COFs before and after loading of RML as well as the corresponding loading capacity.


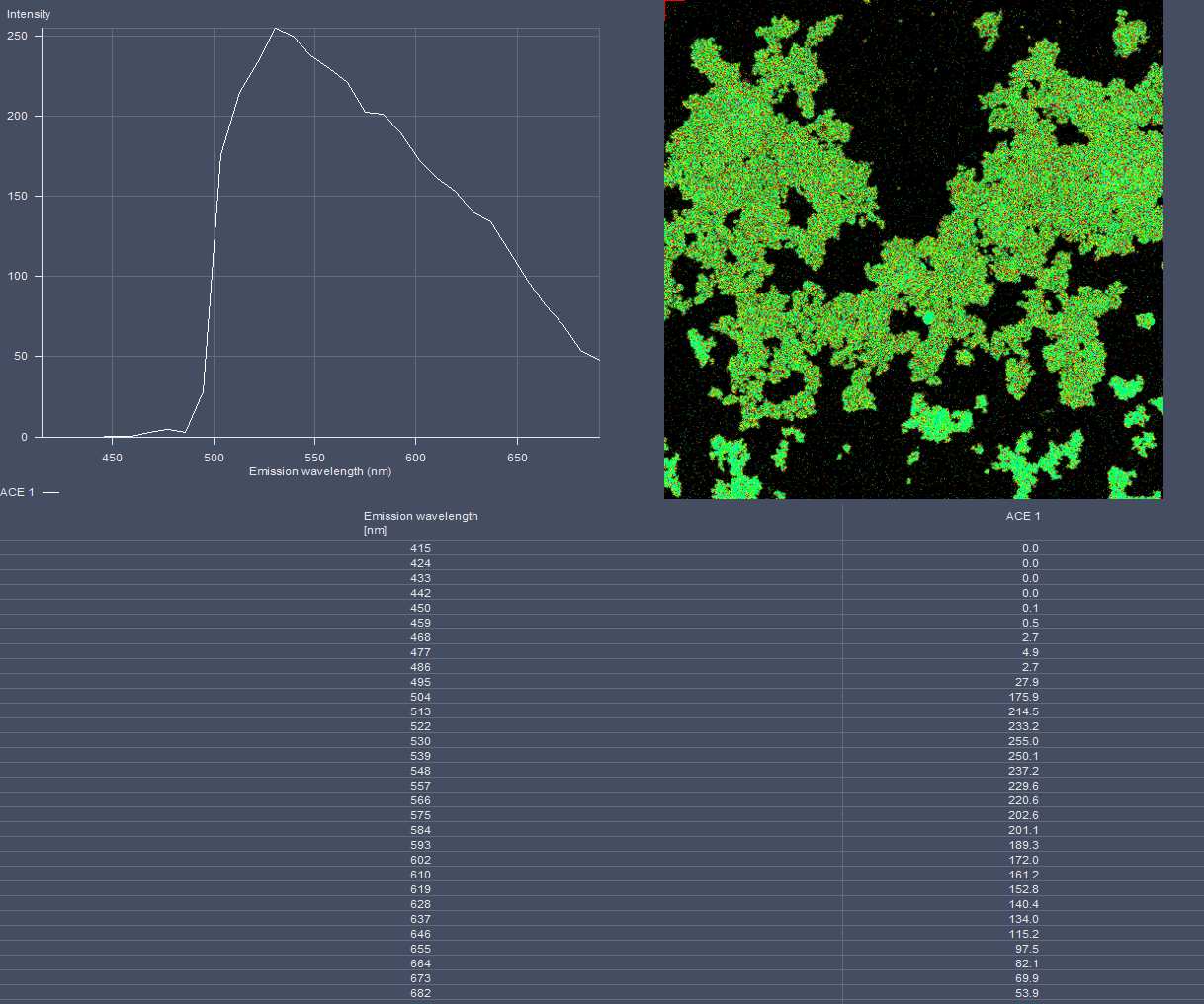

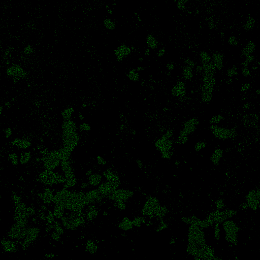

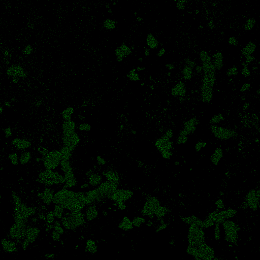

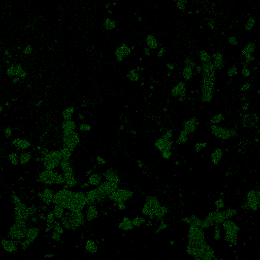

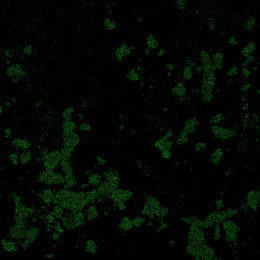

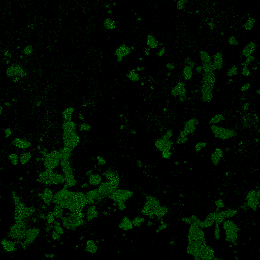

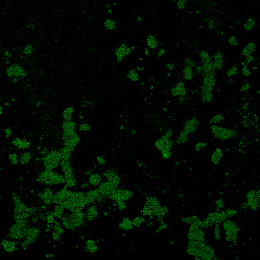

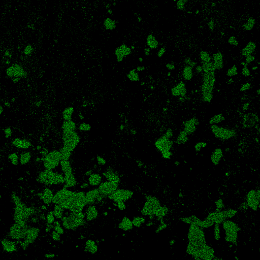

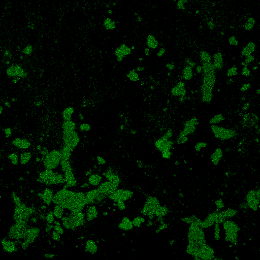

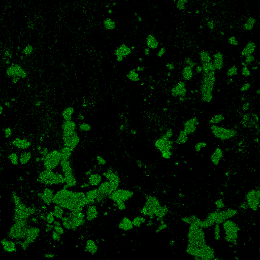

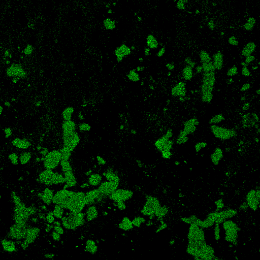

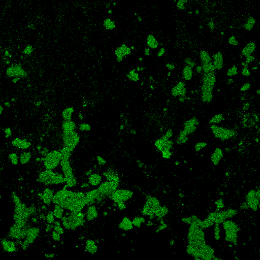

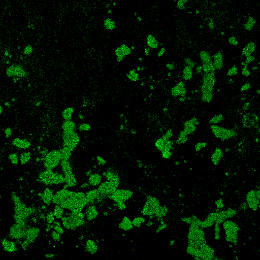

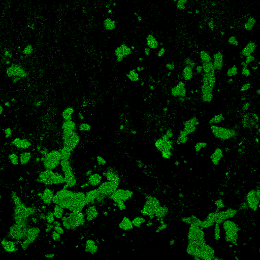

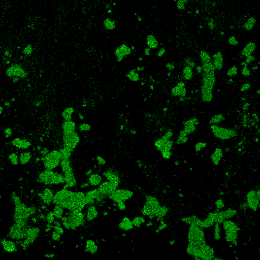

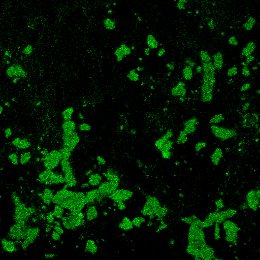

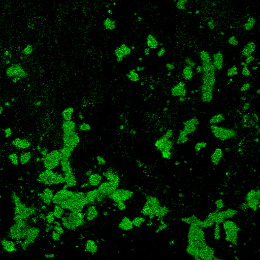

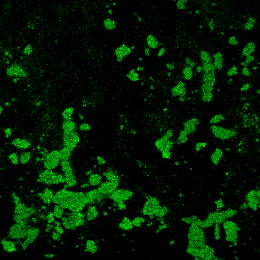

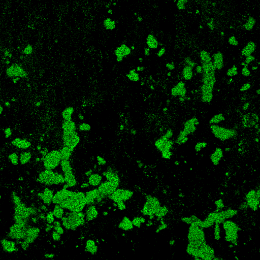

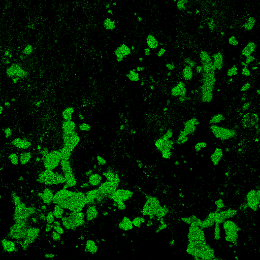

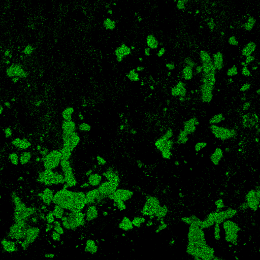

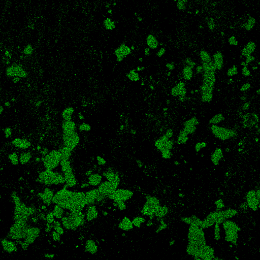

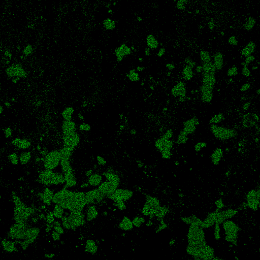

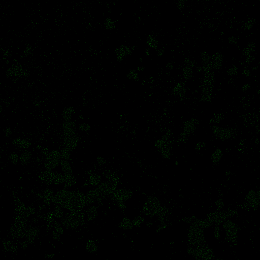

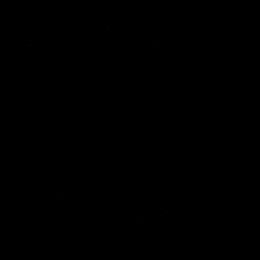

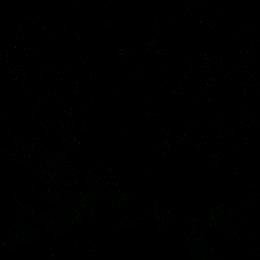

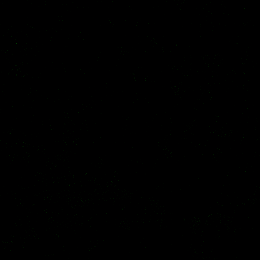

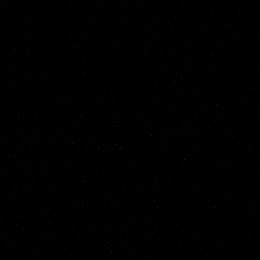

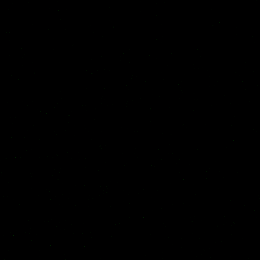

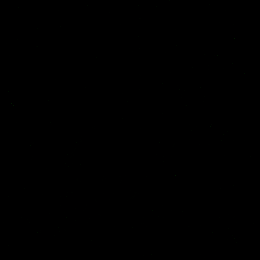

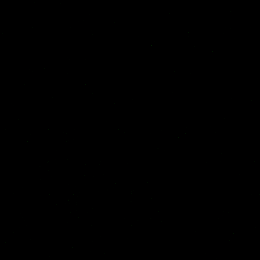

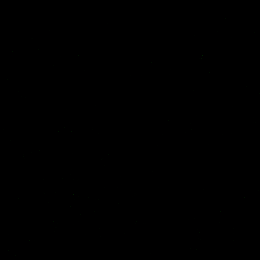

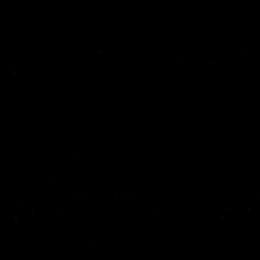

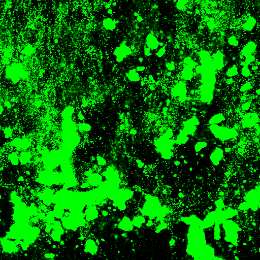


Figure S10. The confocal laser scanning microscope image of Fe_3_O_4_@COF-OMe (excitation at λ=488nm, and long emission at 490-690nm)


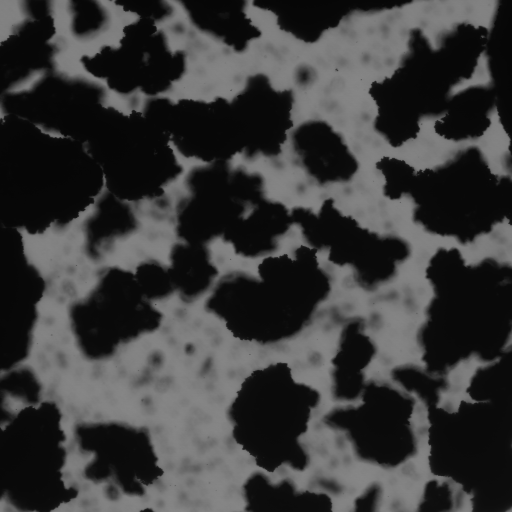

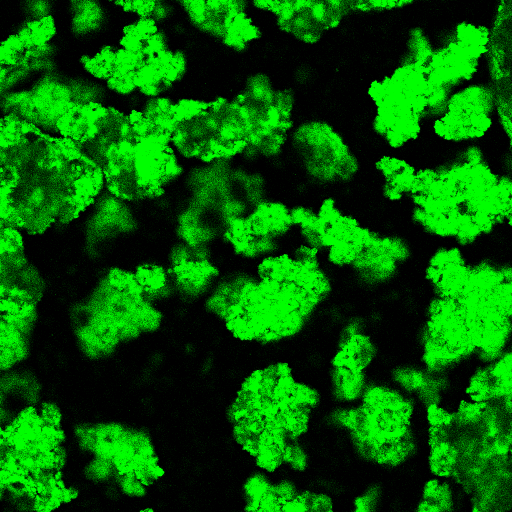

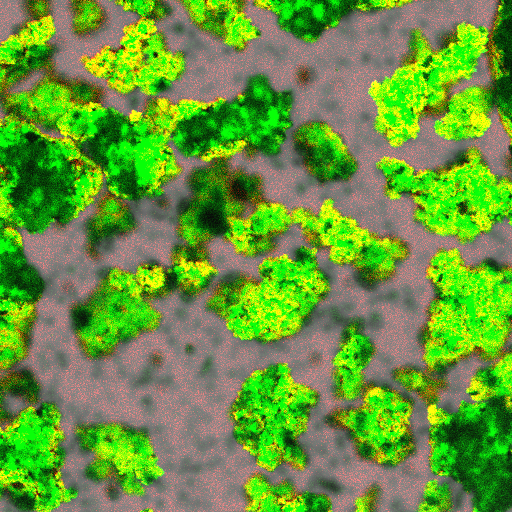

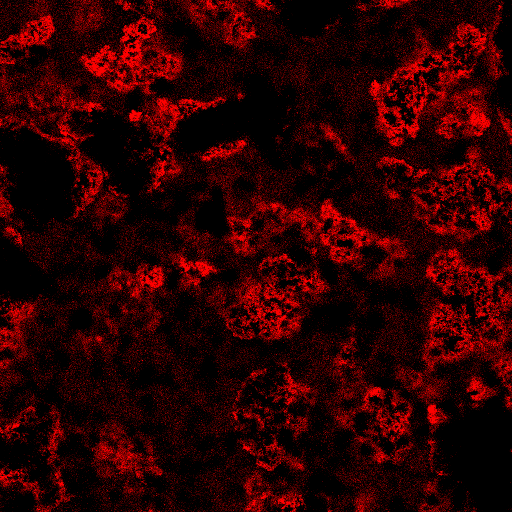


Figure S11. The confocal laser scanning microscope image of RML@Fe_3_O_4_@COF-OMe (excitation at λ=488nm and 543nm).


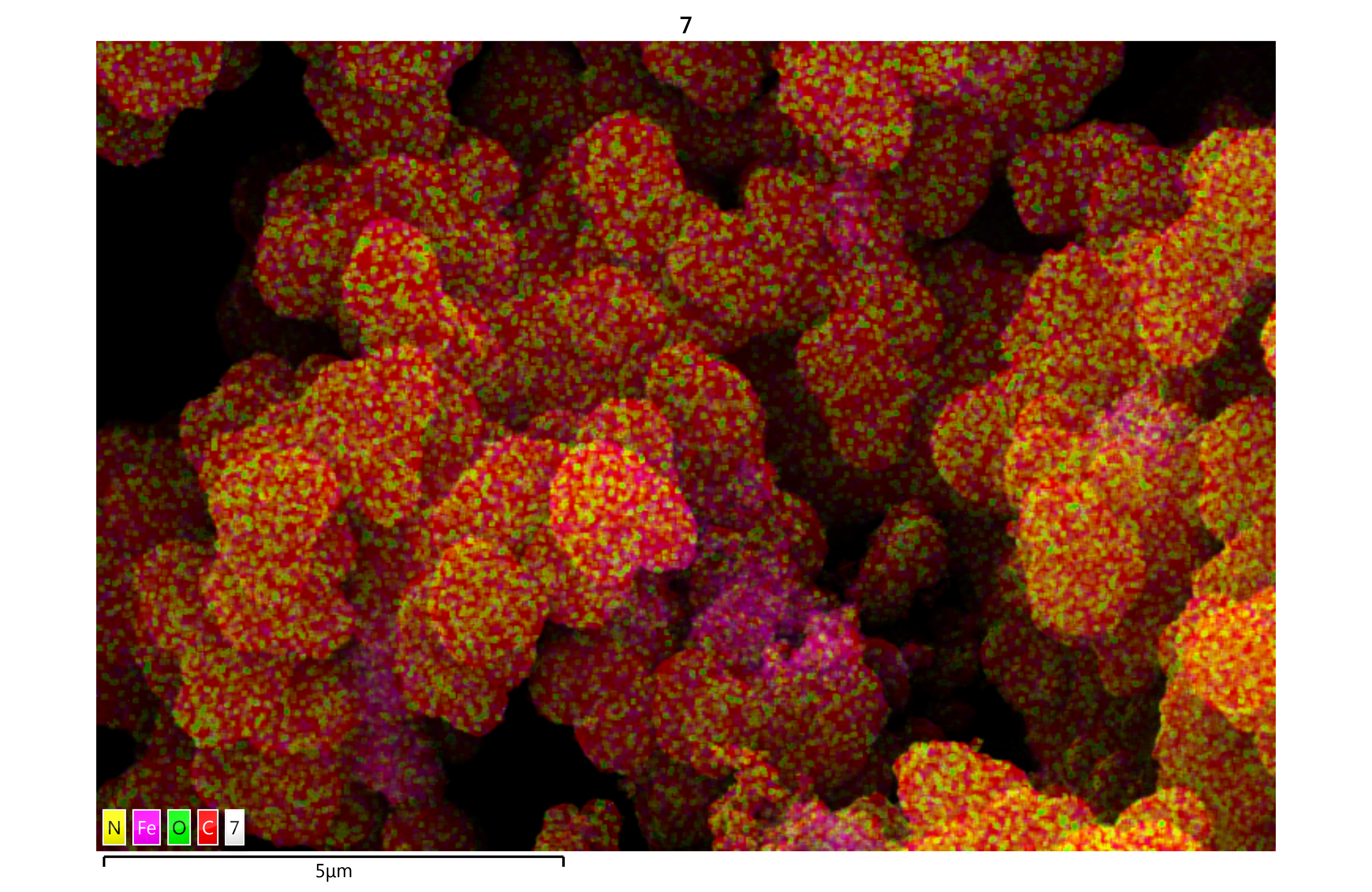

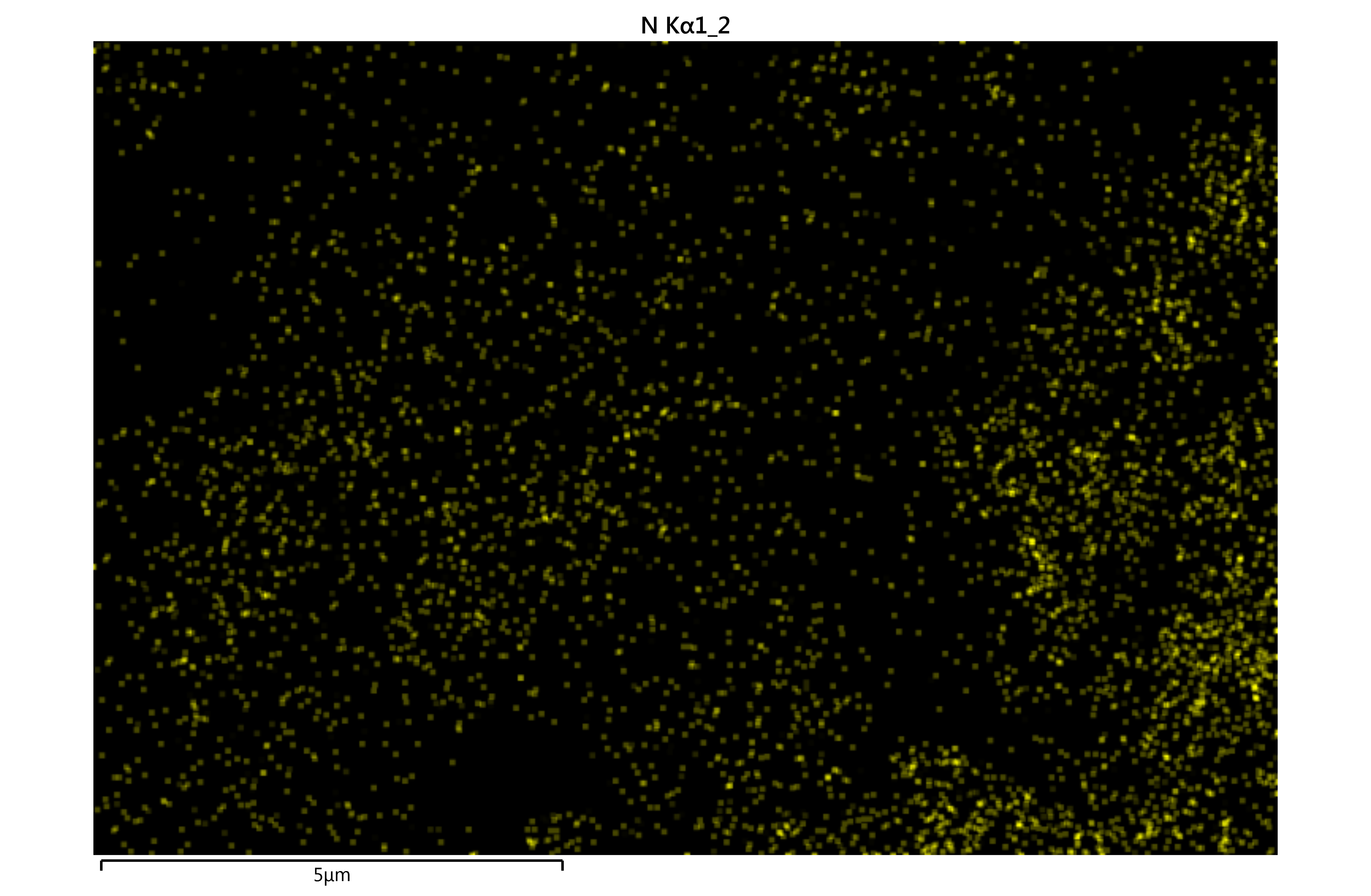

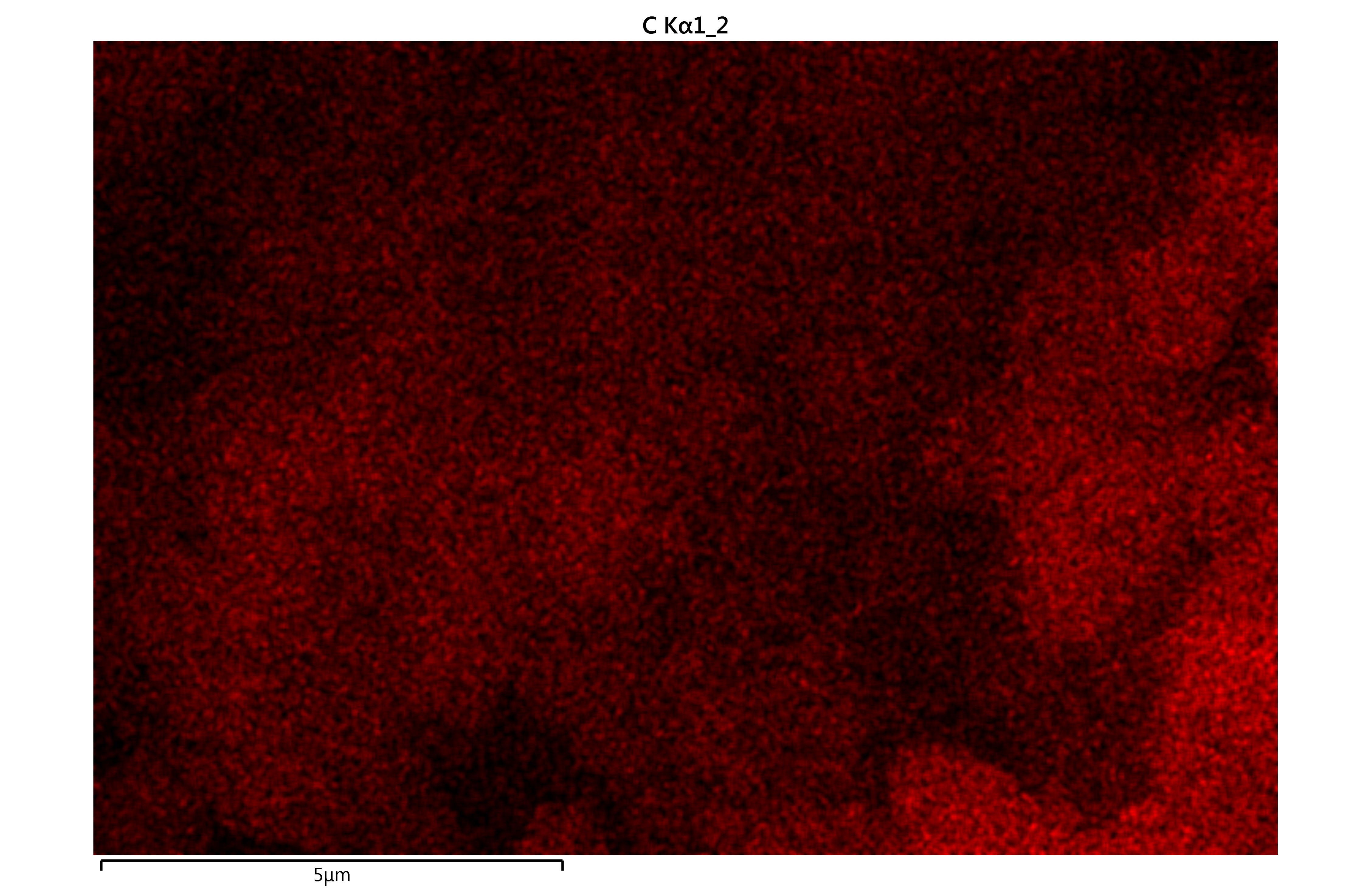

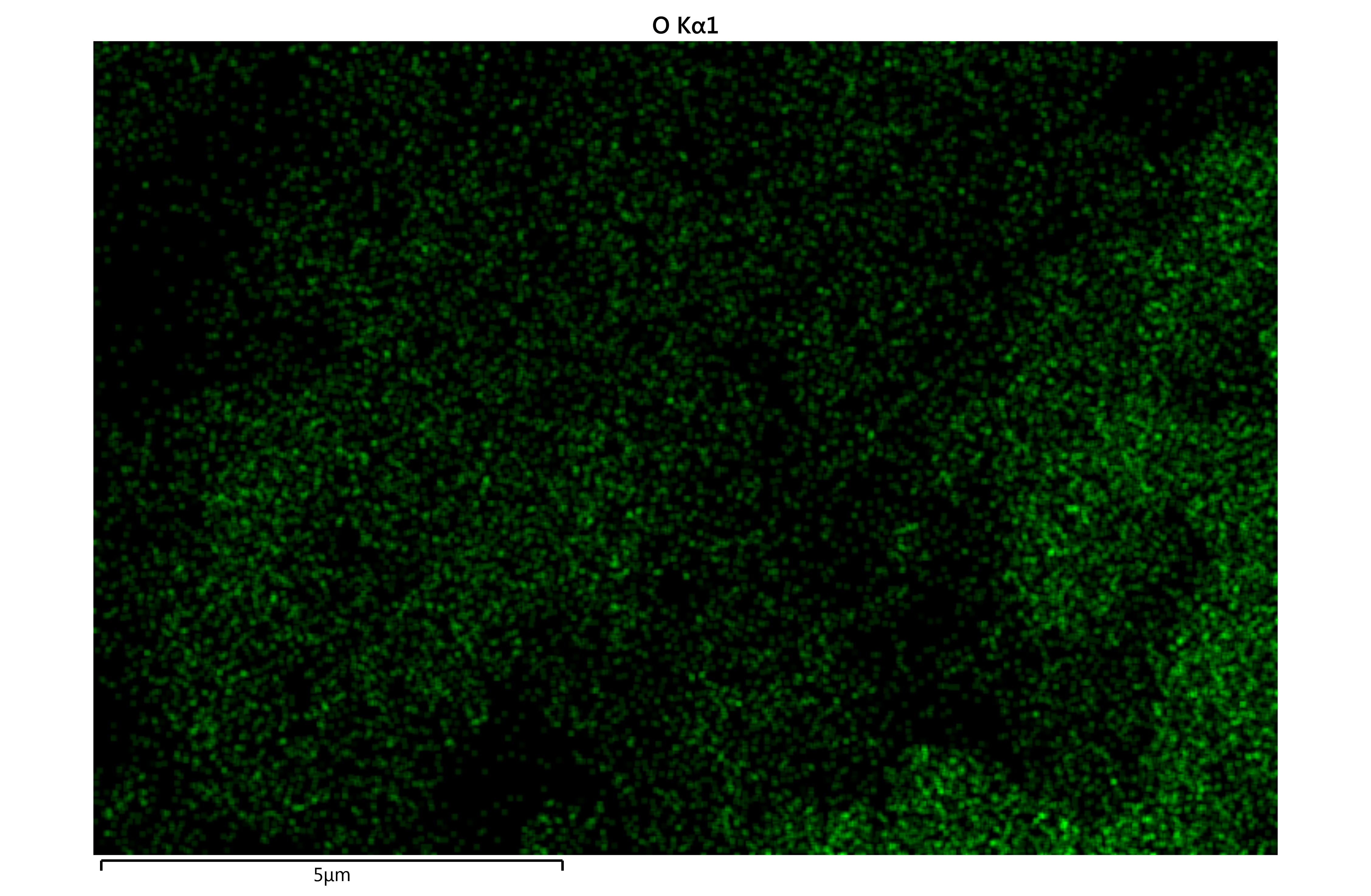

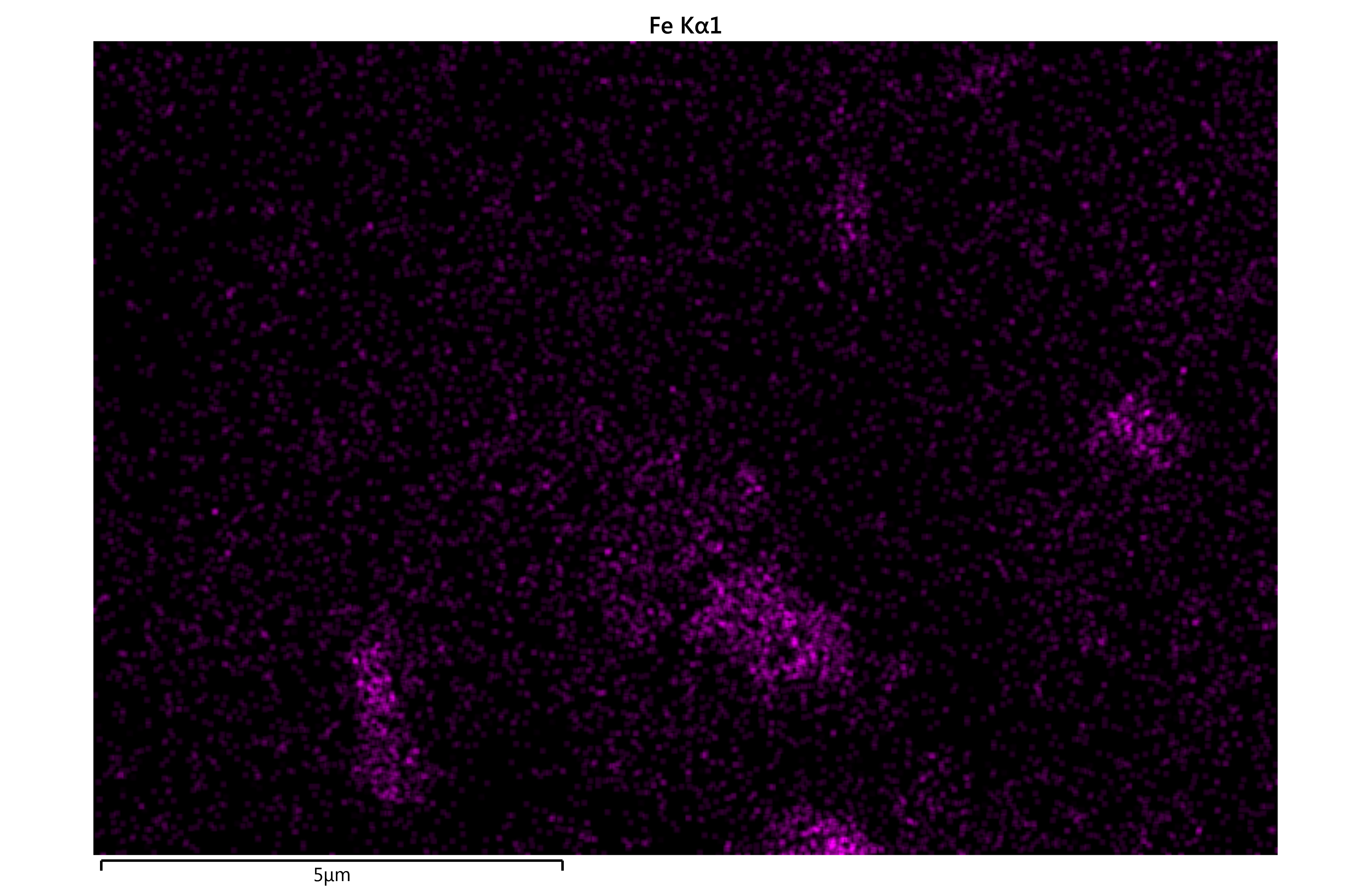
Figure S12. The EDS mapping of Fe_3_O_4_-COF-OMe (N: yellow, C: red, O: green, Fe: purple)


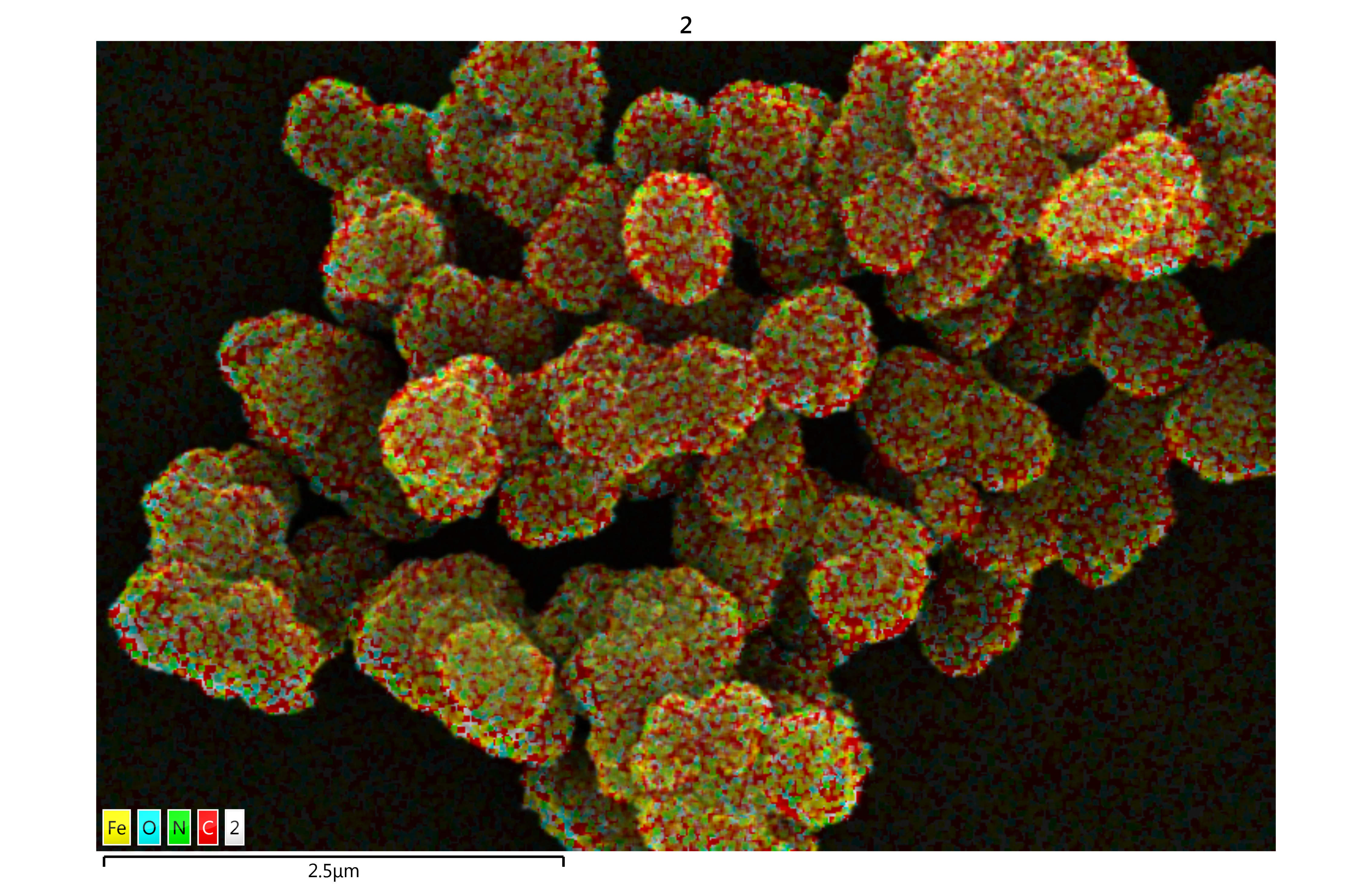

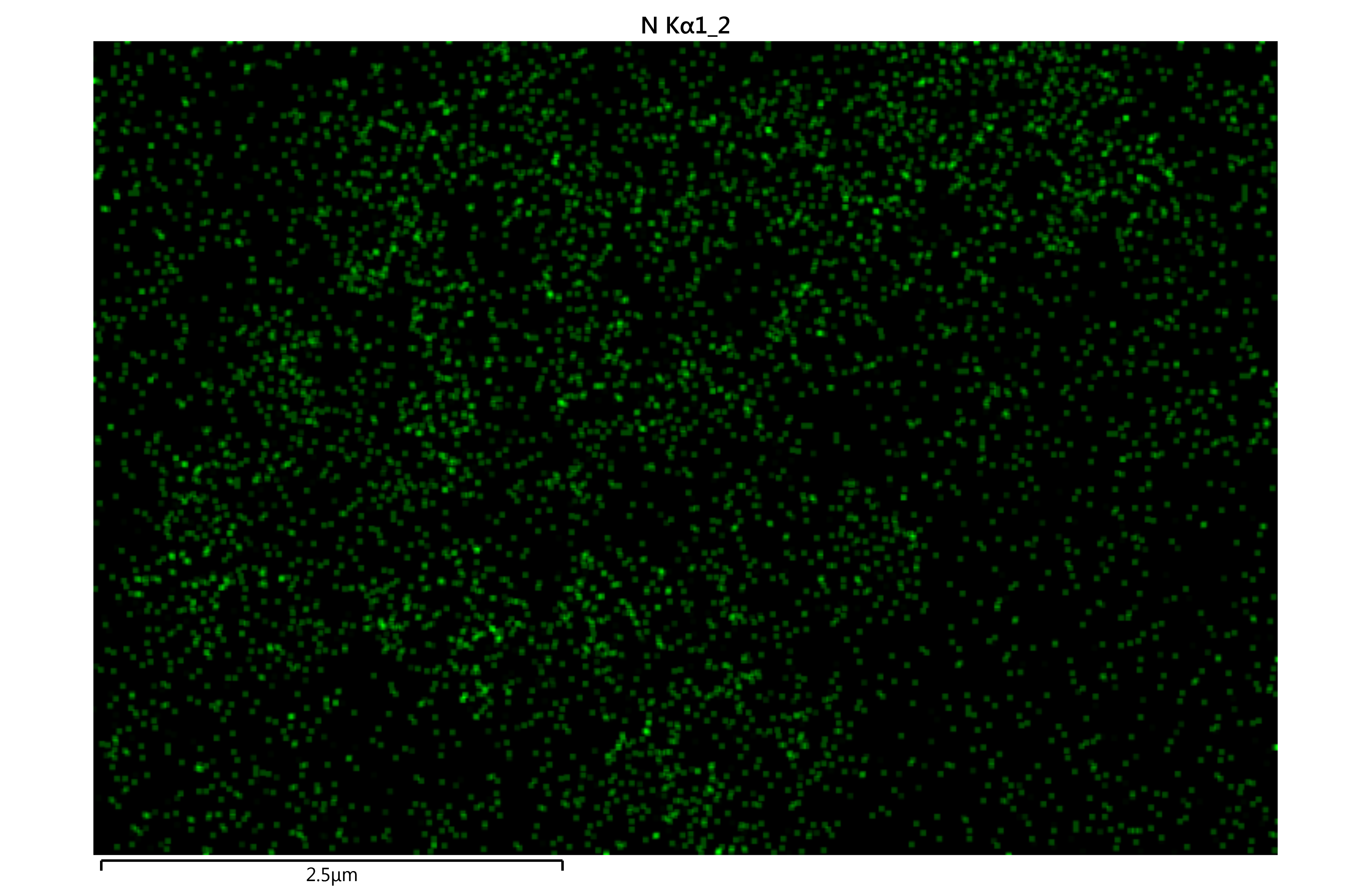

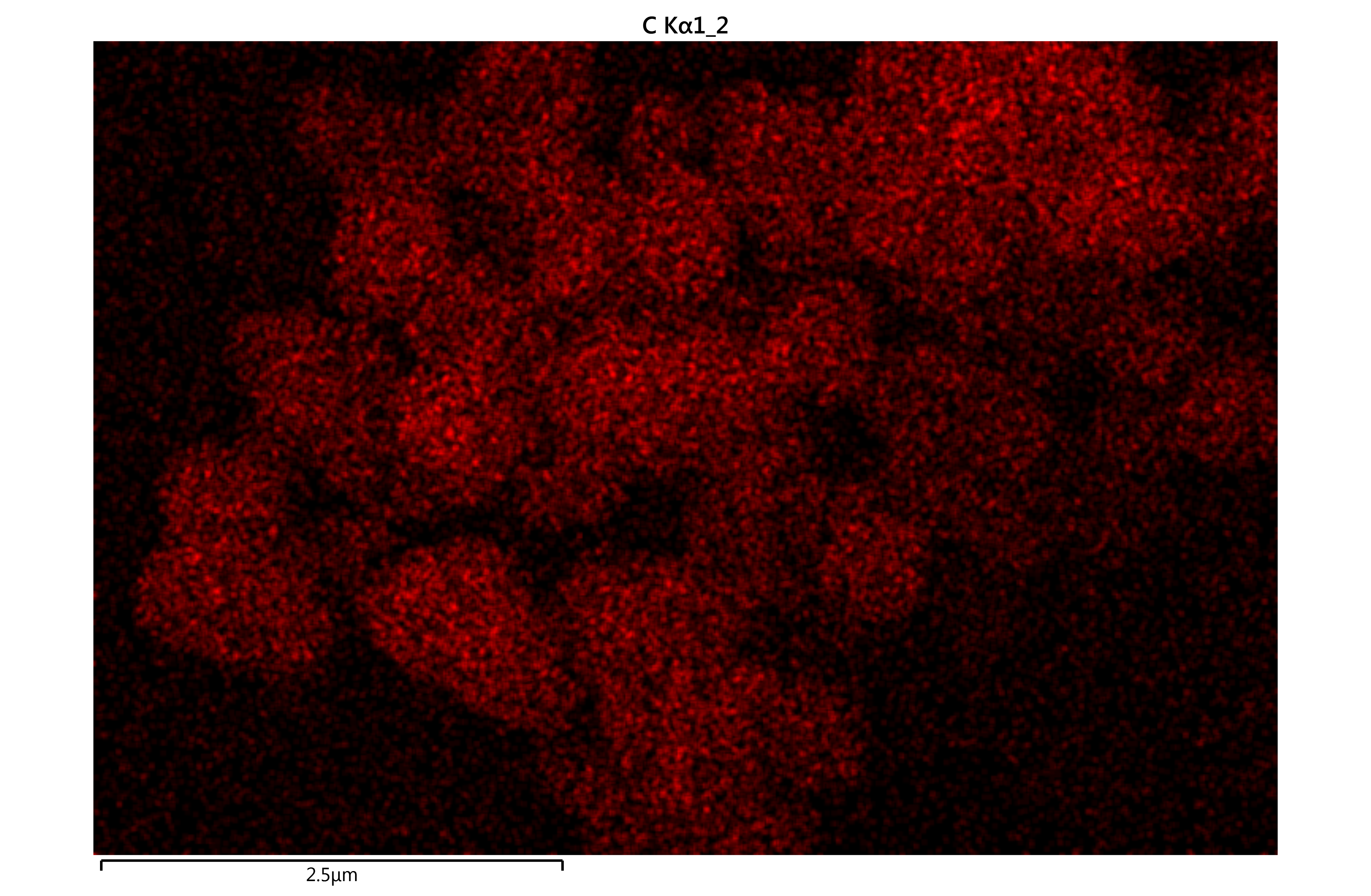

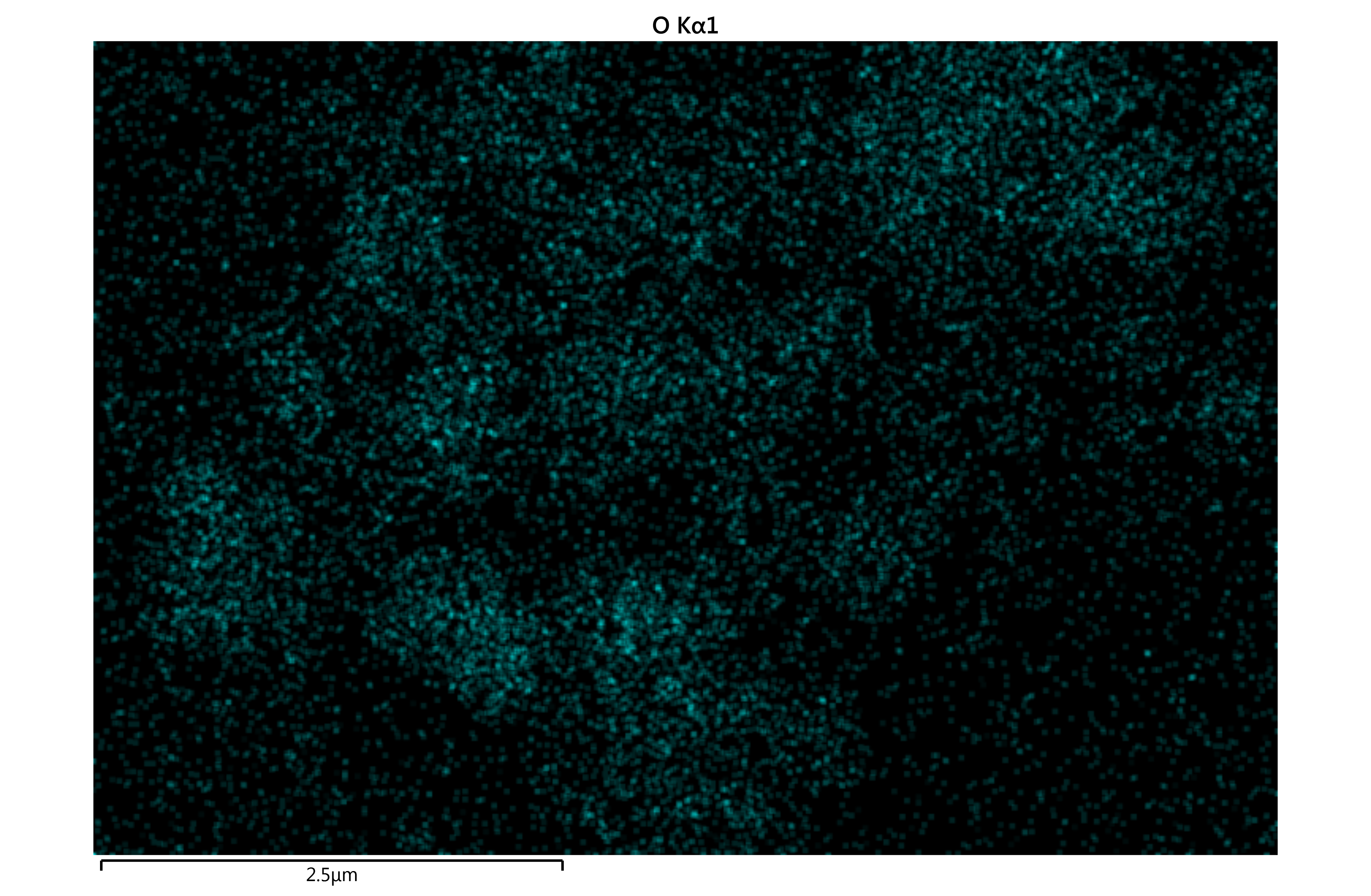

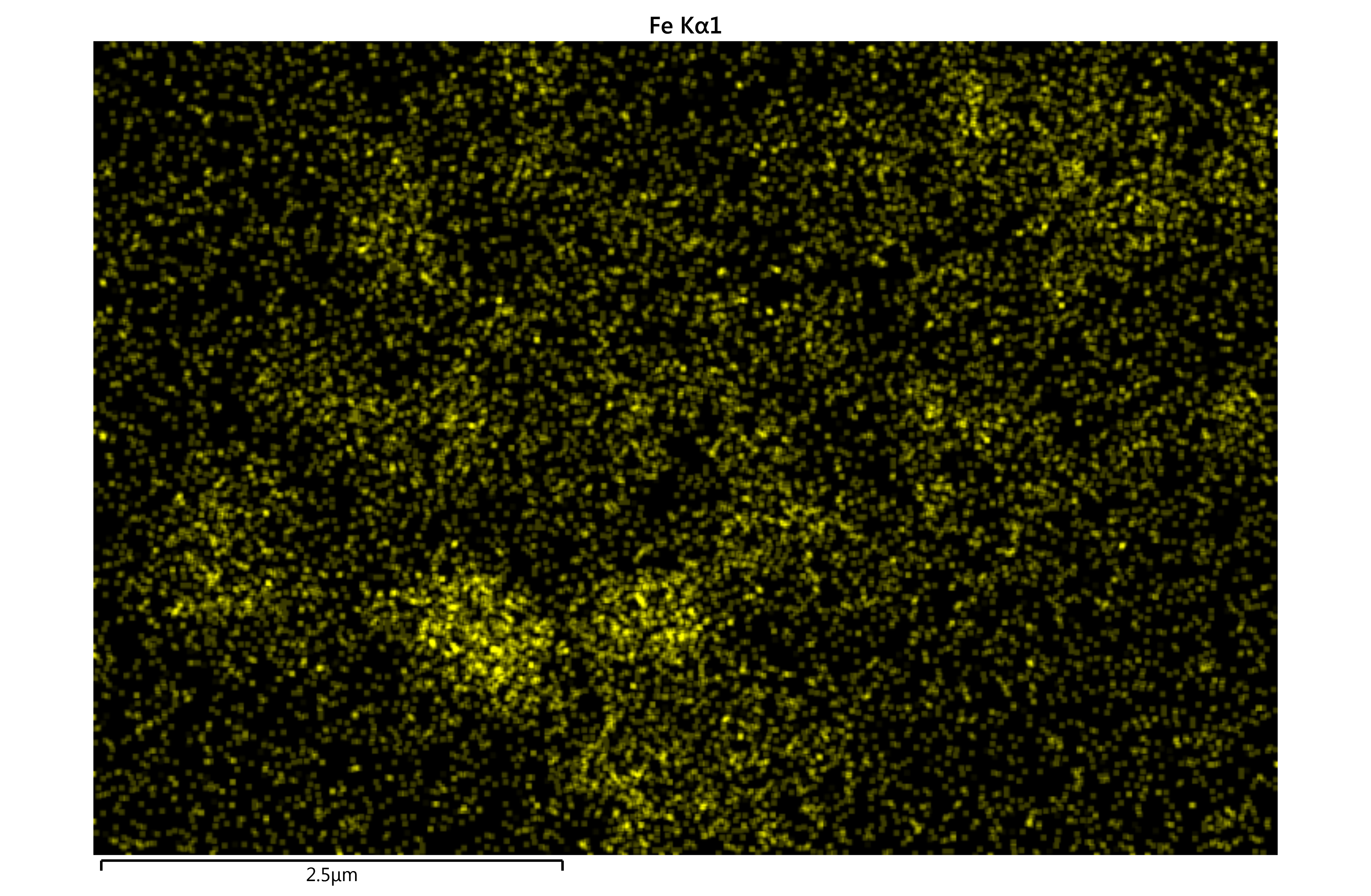


Figure S13. The EDS mapping of Fe_3_O_4_@COF-OMe (N: green, C: red, O: cyan, Fe: yellow)

Figure S14. The transesterification reaction of 2-phenylethanol with vinyl acetate.


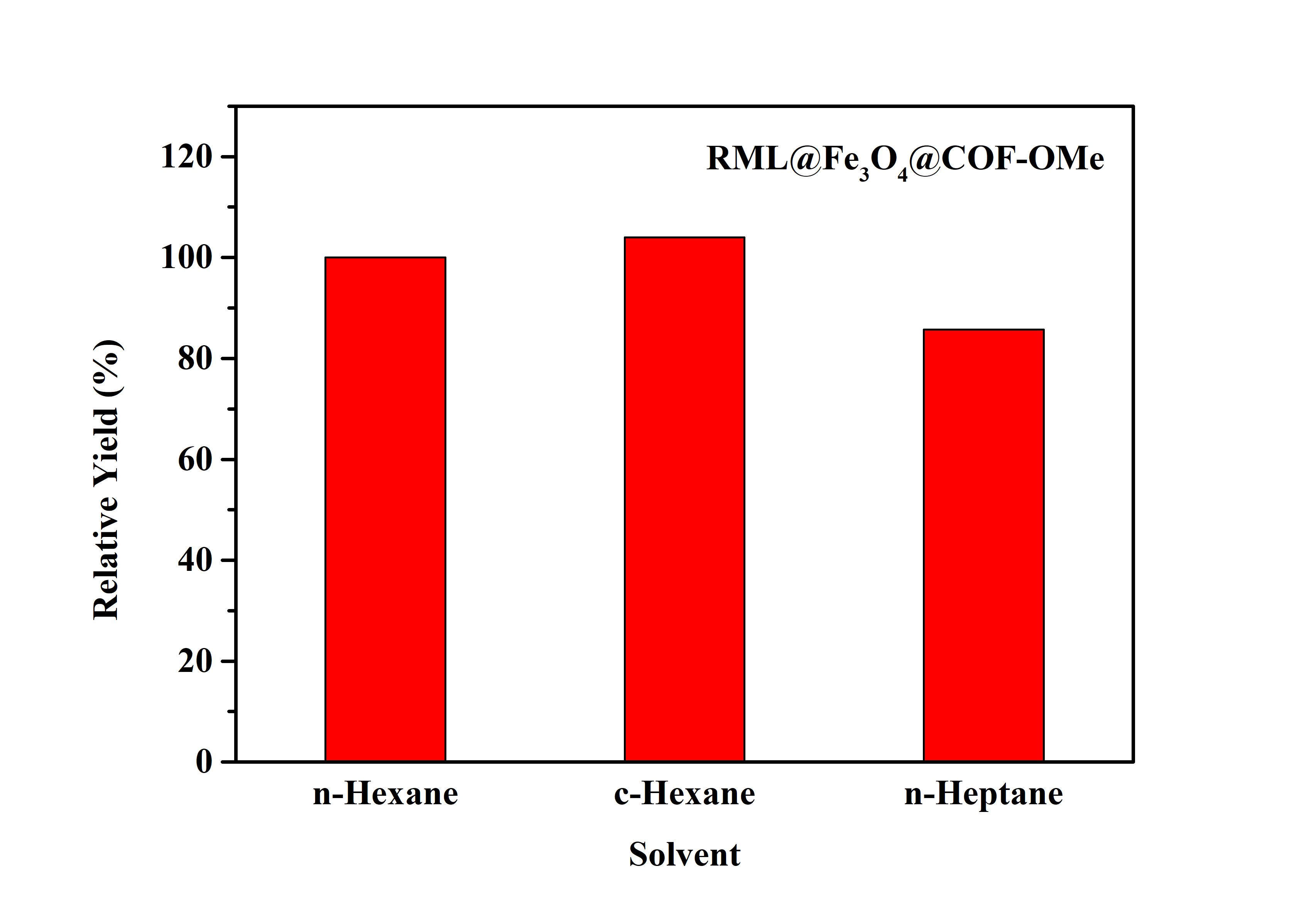
Figure S15. Transesterification yields in different solvents (The yield in n-Hexane is set as 100%).


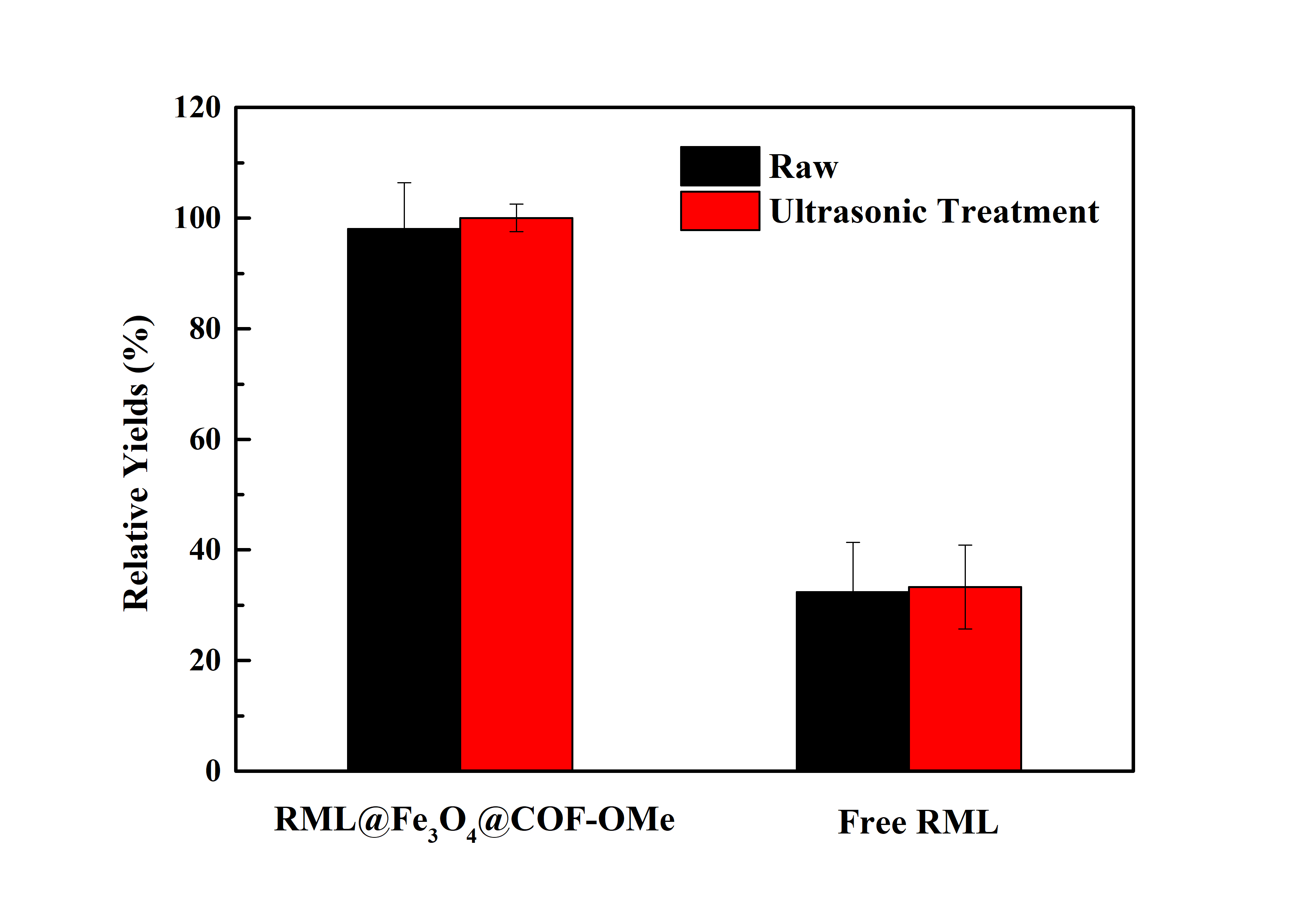


Figure S16. The tolerance of both immobilized RML and free enzyme against ultrasonic operation. The relative yields were displayed by transesterification of 2-phenyl ethanol (20μL) and vinyl acetate (40 μL), performed at 50^o^C, and 150rpm for 24h in n-Hexane.

**Supporting reference**

1. Zhou X-G, Deng P-C, Xv K-L. *Introduction of Spectroscopy*. Beijing: Chemical Industry Press, 2014.
